# Cytokine Imbalance in Schizophrenia. From Research to Clinic: Potential Implications for Treatment

**DOI:** 10.3389/fpsyt.2021.536257

**Published:** 2021-03-05

**Authors:** Marcella Reale, Erica Costantini, Nigel H. Greig

**Affiliations:** ^1^Department of Medical, Oral and Biotechnological Sciences, University “G. d'Annunzio” Chieti-Pescara, Chieti, Italy; ^2^Drug Design and Development Section, Translational Gerontology Branch, Intramural Research Program, National Institute on Aging, National Institutes of Health, Baltimore, MD, United States

**Keywords:** serum molecular target, inflammatory cytokines, T helper type 1, CNS and Immune system cross-talk, molecular targets

## Abstract

Cytokines are one of the most important components of the immune system. They orchestrate the brain's response to infectious and other exogenous insults and are crucial mediators of the cross-talk between the nervous and immune systems. Epidemiological studies have demonstrated that severe infections and autoimmune disorders, in addition to genetic predisposition, are risk factors for schizophrenia. Furthermore, maternal infection during pregnancy appears to increase the risk of schizophrenia, and proinflammatory cytokines may be negatively involved in the neurodevelopmental process. A cytokine imbalance has been described in the blood and cerebrospinal fluid of schizophrenia patients, particularly in the T helper type 1 [Th1] and type 2 [Th2] cytokines, albeit the results of such studies appear to be contradictory. Chronic stress, likewise, appears to contribute to a lasting proinflammatory state and likely also promotes the disorder. The aim of this mini-review is to investigate the roles of different cytokines in the pathophysiology of schizophrenia and define how cytokines may represent key molecular targets to regulate for the prevention and treatment of schizophrenia. How current antipsychotic drugs impact cytokine networks is also evaluated. In this context, we propose to change the focus of schizophrenia from a traditionally defined brain disorder, to one that is substantially impacted by the periphery and immune system.

## Introduction

Schizophrenia, a chronic and often debilitating mental disorder, impacts ~1% of the world's population and generally occurs in late adolescence or early adulthood. It is characterized by a symptomatology that includes the presence of auditory, visual, tactile and olfactory hallucinations, delusions, confusion, impaired concentration that includes cognitive scarcity, lethargy, and movement disorders (1). Although genetic vulnerability and environmental stressors during the early stages of life are fundamental for the progression of schizophrenia, inflammation is considered a primary causative/contributing/mediating factor in the onset of schizophrenia (2–4). Disturbances of the immune system and its complex interactions with the nervous system may hence contribute to the pathogenesis and pathophysiology of schizophrenia (5, 6). As a consequence, a bidirectional interaction between the immune system and the brain has aroused an increasing interest in the role of the immune system in neuropsychiatric diseases. Abnormal blood lymphocyte parameters, such as the numbers of total T lymphocytes, T helper cells, an increased CD4/CD8 ratio (7), and a decreased mitogen-induced lymphocyte proliferation have been reported (8), and the presence of select antibrain antibodies have been detected in the serum of schizophrenic patients (9).

An interesting current debate concerns the relationship between different immune factors and the pathophysiology of schizophrenia, and particularly cytokine dysregulation in patients with schizophrenia. It is well-known that cytokines are key messengers in the cross-talk between the central nervous system (CNS) and immune cells. In the light of this, several studies have hypothesized that in patients with schizophrenia and severe mood disorders, the proinflammatory cytokine microenvironment is likely involved in the pathogenesis and pathophysiology of schizophrenia and the ensuing psychopathological symptoms (10–14).

An increasing number of studies indicating a role of inflammation and immunity in the pathogenesis of symptoms of schizophrenia have provided evidence that systemic inflammation can exert a profound influence on the brain that leads to changes in mood, cognition, and behavior. In this regard, the peripheral immune system-to-brain communication pathways have been studied extensively in the context of other neuroinflammmatory diseases in which inflammatory cytokines are, likewise, considered to play a critical role (15–17). Several hypotheses have been formulated to both highlight and account for the involvement of immune cells and cytokines in schizophrenia. One hypothesis was based on the macrophage-T lymphocyte theory, according to which cytokines such as interleukin-1 (IL-1), IL-2, tumor necrosis factor-α (TNF-α), interferon-α (IFN-α), and IFN-γ that are produced by chronically activated macrophages and T lymphocytes, are the key mediators of schizophrenia (18). A further hypothesis is the Th2 hypothesis, according to which Th2-mediated immune responses are the most important events in schizophrenia (19). There is evidence to indicate that altered cytokines and gray matter abnormalities, such as the cortical thickness of the bilateral Broca's area and temporal gyrus, are associated with schizophrenia (20). In this light, it is possible to speculate that abnormal levels of cytokines could potentially be used as a disease indicator and may provide a diagnostic or prognostic biomarker in schizophrenia (21, 22).

## Road Map

The focus of this mini-review is to evaluate whether and how alterations in peripheral cytokines may impact schizophrenia. In undertaking this task, we first define what cytokines are and where they derive from. We next review their involvement in schizophrenia, particularly in relation to studies that have quantified elevations and declines of select cytokines in the periphery or CNS. In this regard, there are numerous studies providing data on different cytokine combinations, with several providing apparently conflicting results in relation to change direction that potentially arise from disease stage and patient state. Medications may, likewise, modify cytokine levels, and we therefore review studies assessing them in subjects treated with antipsychotics. Although first-line therapy, numerous patients do not adequately respond to antipsychotics, and we hence evaluate studies incorporating other medications with a focus on immunomodulatory agents that more directly impact cytokine levels. Finally, we provide a synopsis of where the field is heading and how recent advances, such as sampling exosomes enriched for brain origin, may aid our understanding of cytokine imbalances in schizophrenia and their targeting as a treatment approach.

## Cytokine Overview

Cytokines, low molecular weight proteins, affect nearly every biological activity, including embryonic development, disease pathogenesis, specific and non-specific immune responses, cognitive function, as well as progression of the degenerative processes of aging. These effects are mediated by impacting the cells that secrete them (i.e., autocrine action), neighbor them (i.e., paracrine action), and/or are remote from them (i.e., endocrine action). In this regard, the same cytokine can act on many different cell types (i.e., pleiotropic action), or a similar function can be instigated by different cytokines and the same cytokine may have overlapping actions and may regulate several different immune functions (i.e., redundant action). Cytokines can also act synergistically or antagonistically by activation or inhibition of its target cells to generate additional cytokines to, thereby, amplify or dampen an inflammatory response. Although cytokines are generated by numerous cell types, such as fibroblasts and endothelial and epithelial cells, the predominant producers are the white blood cells and, in particular, T cell subsets and macrophages.

In this regard, CD4+ T helper cells are traditionally considered the cytokine-generating cells and, according to their pattern of cytokine production, Th cells are classified as Th1, Th2 (23), Th17 (24), and Th9 cells (25). In addition, two subpopulations of CD8+ effector T cells; specifically, types 1 and 2 cytokine-producing subsets have been identified (26) and, in both physiological and pathological processes, cytokines may be produced in and by peripheral nerve tissue by resident and recruited macrophages, mast cells, endothelial cells, as well as Schwann cells.

Cytokines are classified as proinflammatory and anti-inflammatory. The time-dependent pro- and anti-inflammatory balance determines the outcome of an inflammatory response. As an example among many, elevated levels of proinflammatory cytokines, such as IL-6, IL-1, IL-17, and TNF-α have been demonstrated in the cerebrospinal fluid (CSF) and in demyelinating plaques of patients with multiple sclerosis (MS), suggesting a pivotal role in the pathogenesis of this and other neurodegenerative disorders (27, 28). The synthesis and release of cytokines in response to a variety of stimuli supports their interaction/binding to receptors that, in turn, signal a response to their target cells—whether proximate or distant—that then leads to a change in cell function or activity. Such cytokine receptors are linked to multiple signaling pathways within the cytoplasm and nucleus, leading to transcriptional and post-transcriptional activation of multiple factors. In this regard, select transcription factors, such as nuclear factor κB (NF-κB), activator protein-1 (AP-1), and nuclear factor of activated T cell (NFAT), are crucial in cytokine production (Table 1).

**Table 1:** Several cytokines, cytokine receptors, signaling, and source.

**Cytokine**	**Receptor family**	**Signaling**	**Source**
IL-1	Immunoglobulin superfamily receptors	NF-κB, IRAK, MyD88, TRAF6	Many cells, expecially monocytes/macrophages; epithelial and endotelial cells; fibroblasts; astrocytes
IL-2	Class I cytokine receptors	JAK1, JAK3, STAT5	Th1, NK cells
IL-4	Class I cytokine receptors	JAK1, JAK3, STAT6	Th2, mast cells, other cells
IL-6	Class I cytokine receptors	JAK1, STAT3	Macrophages, fibroblasts, T cells
IL-10	Class II cytokine receptors	JAK1, TYK2, STAT3	Th2, Treg
IL-12	Class I cytokine receptors	JAK2, TYK2, STAT4	Macrophages, NK cells, DCs, B cells
IL-17	Immunoglobulin superfamily receptors	MAPKs, PI3K, NF-κB.	Th17
IL-18	Immunoglobulin superfamily receptors	IRAK, MyD88, TRAF6, NF-κB	Many cells, expecially macrophages, keratinocytes
IFNγ	Class II cytokine receptors	JAK1, JAK2, STAT1	Th1, NK cells
TNFα	TNF receptors	NF-κB; JNK, ERK, p38	Activated myeloid, T and other cells
TGFβ	Receptor serine kinase family	MAPK	Treg, macrophages, other

The expression level of receptors can be induced by some cytokines and may, thereby, modify target cell responsiveness. As a consequence, cytokine interactions can be considered a “cytokine network” in light of the numerous potential feedback mechanisms between the various cytokines as well as their targets. Depending on the cytokine concentrations available, target and receptor expressions, the activation of different signal transduction pathways occurs that will lead to a different gene expression in response to different cytokines that share some biological effects. This adavantagiously provides the potential combinations of cytokines to orchestrate multiple different actions to maintain homeostasis in response to a broad array of challenges during health, but, conversely creates a complex picture that is difficult to interpret in the presence of disease and, particularly a mental disorder such as schizophrenia. Furthermore, different secretory pathways characterize the cytokine secretion process based on a cytokine's function and cell type. In this regard, many immune cells store cytokines in granules that allow their rapid release in response to receptor signaling from toll-like receptors (TLRs), Fc receptors, cytokine receptors, and complement receptors, among others (29, 30). Secretion may occur *via* the constitutive or non-conventional secretory pathways, with cytokines such as IL-2, IL-3, IL-6, IL-10, IL-12, and TNF-α being constitutively secreted, while others such as IL-1β, IL-1α, IL-33, and high-mobility group box 1 (HMGB1) being unconventionally secreted.

In synopsis, the synthesis and release of cytokines is an important part of the immune response and can act in a homeostatic protective manner or, when secreted inappropriately or excessively, can be involved in chronic conditions such as a generalized systemic inflammatory response or a neurodegenerative/neuropsychiatric disorder. As cytokines have such potent actions, it has become increasingly clear that dysregulation of cytokine generation and release, as well as cytokine signaling can contribute to human disease and lead to pathogenic effects.

## Cytokines in Schizophrenia

**Figure 1:**
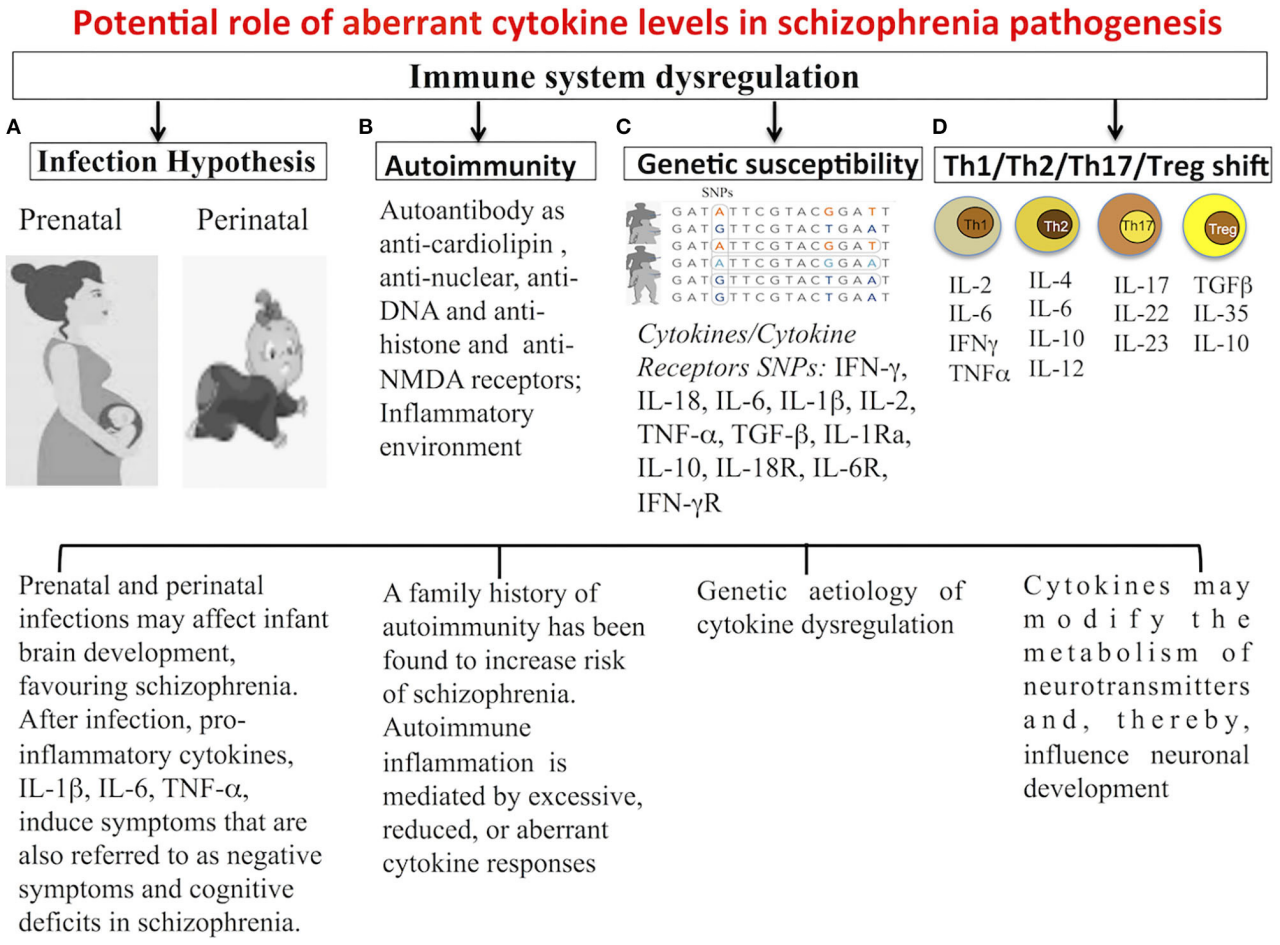
Potential role of aberrant cytokine levels in schizophrenia pathogenesis. Cytokines may represent a common pathway for environmental and genetic components of schizophrenia: **(A)** Cytokines produced after immune activation due to prenatal or perinatal infection may contribute to schizophrenia. **(B)** Autoantibody, dysregulated T cell polarization and inflammatory environment, detected in autoimmune diseases, were associated with an increased risk of psychotic disorders and vice versa. **(C)** Cytokine alteration might be genetically determined and contributed to the risk and pathogenesis of schizophrenia. **(D)** Alterations of Th1/Th2/Th17/Treg balance influence the dopaminergic, noradrenergic, and serotonergic neurotransmission.

Our increasing understanding of the functioning of the immune system has strengthened psycho-neuroimmunological theories hypothesizing that schizophrenia is a systemic syndrome, involving both the nervous and immune systems, in which abnormalities in immune system and cytokine functioning have a pivotal role (Figure 1). Recently, evidence linking schizophrenia to autoimmunity has been highlighted; autoimmune-related antibodies anticardiolipin, antinuclear, anti-DNA, antihistone, and anti-NMDA receptors have been reported present in the serum of schizophrenia patients. Gene polymorphisms of several cytokines are associated with the development of the schizophrenia syndrome. In patients with schizophrenia, presence of gene polymorphisms of proinflammatory cytokines, such as IL-1β and IL-6, have been linked to high serum levels of these cytokines. An emerging literature suggests that prenatal and postnatal exposure to pathogens may contribute to the etiopathogenesis of schizophrenia *via* the actions of cytokines. In fact, cytokines produced in response to infection are not only involved in the inflammatory response but also in the development and function of the CNS. During prenatal infections, maternally produced cytokines may cross the placenta and blood-brain barrier and drive behavioral, neurochemical, psychophysiologic, and histologic abnormalities ultimately found in schizophrenia patients (31). An imbalance between T helper (Th) 1, Th2, Th17, and Treg cells and cytokines produced, represent the essential component of immune dysregulation in schizophrenia.

### Peripheral Cytokines

Numerous studies have investigated alterations in peripheral cytokine levels in schizophrenia. Among these, the study of Smith and Maes proposed that in schizophrenia, chronically activated macrophages and T lymphocytes produce cytokines, such as TNF-α, IL-1, IL-2, IFN-α, and IFN-γ, that have a key role in this disorder's development (18). On the basis of this study, the relationship between schizophrenia and cytokine levels was evaluated, and two meta-analyses were performed to shed light on the relationship of abnormal cytokine levels with schizophrenia. Data from 62 studies, analyzed IFN-γ, IL-4, IL-2, soluble IL-2 receptor (sIL-2R), IL-1β, IL-1 receptor antagonist (IL-1RA), TNF-α, IL-6, soluble IL-6 receptor (sIL-6R), and IL-10 in schizophrenia. Increased levels of IL-1RA, sIL-2R, and IL-6 were evident *in vivo*, whereas *in vitro* IL-2 was decreased, and no significant differences were observed for the other cytokines. This, thereby, provided the first evidence to consider the occurrence of an inflammatory syndrome in schizophrenia (32). A meta-analysis by Miller et al. (33) investigated cytokines in schizophrenia at various disease stages and treatment conditions in an analysis of 40 studies. Patients were separated into three groups: drug-naïve first-episode psychosis, acute relapse of psychosis, and schizophrenia patients who were stable medicated outpatients with treatment-resistant psychosis. Results showed that patients with first-episode psychosis and those with acute relapse of psychosis had significantly elevated levels of IL-1β, IL-6, TNF-α, IFN-γ, and IL-12, and patients under antipsychotic treatment showed a significant decline in IL-6, IL-1β, and IFN-γ and a rise in IL-12 and soluble IL-2 receptor.

**Figure 2:**
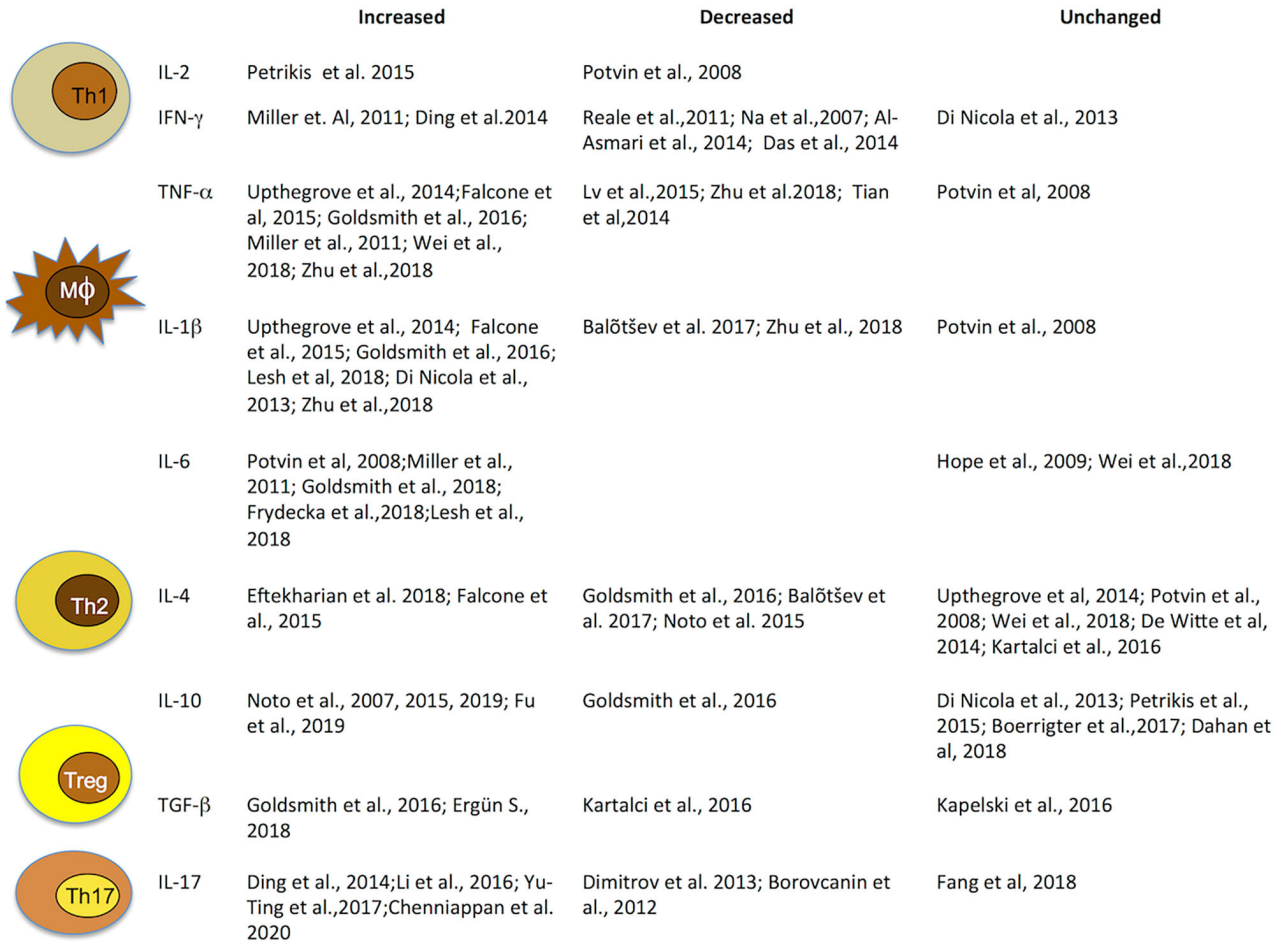
Immune cells and related cytokines. Immune cells and related cytokines that modulate immunity. Level variation of most investigated cytokines implicated in schizophrenia. IFN, interferon; IL, interleukin; TGF, transforming growth factor; TNF, tumor necrosis factor.

Taken together, these findings highlight that cytokine alterations in schizophrenia may vary with clinical status and antipsychotic treatments. Thus, in light of significant heterogeneity across studies, due to patient numbers, different methods of cytokine measurements, use of different diagnostic systems and factors such as age, gender, and smoking habits, concomitant infectious, endocrine, or cardiovascular diseases and obesity, results are highly heterogeneous and report elevated, decreased, as well as unaltered levels of cytokines. Hence, they must be interpreted with caution (Figure 2).

Numerous cytokines, particularly IL-1β, IL-6, IL-18, and TNF-α, are released from microglia and astrocytes and can be quantified in brain, CSF, and plasma. Whereas the CNS is generally considered sheltered from the peripheral immune system by the blood-brain barrier (BBB), serum cytokines can reach the CNS under normal physiological conditions; for example, by saturable transport (34, 35) or BBB damage (36) or *via* the circumventricular organs that lack a classical BBB (37), and thus a peripheral contribution to CSF cytokine levels should not be excluded.

### CNS Cytokines

Largely due to the lack of a matched control group of healthy volunteers, relatively few studies have analyzed differences in the level of cytokines within CSF, and only a restricted number of cytokines have been evaluated. In the CSF of patients with drug-naïve schizophrenia, levels of IL-1β were found elevated (38), whereas levels of IL-6 and IL-8 were reliably detectable and unchanged, and levels of IL-2, IL-4, IL-5, IL-10, granulocyte–macrophage-colony stimulating factor, IFN-γ, and TNF-α were at the level of detection and appeared unchanged. In contrast, levels of IL-1β were found reduced in the CSF and serum of treated schizophrenic patients, compared with healthy controls, IL-2, IL-6, and TNF-α were determined unchanged, and soluble IL-2 receptor was found decreased in CSF and highly elevated in serum Barak et al. (39).

Although the exact role of cytokines in schizophrenia and the correlation between clinical status, disease duration, symptom gravity, and cytokine remain to be fully clarified, evaluation of their level variations in CSF and plasma offers further support to an immunological component in schizophrenia pathogenesis (Table 2).

**Table 2:** Relationship between levels of key cytokines and severity of clinical symptoms.

**High**	
IL-6 and lL-4	Longer disease duration
IL-6 and IL-1β	More severe positive symptoms
IL-6, TNF-α, IL-1β, IFN-γ, IL-4, TGF-β	Exacerbated negative symptoms
IL-6, IL-17, TGF-β	Increased PANSS score
IL-6	Worse cognitive abilities
**Low**	
IL-2, IL-17	Exacerbated negative symptoms
TNF-α and IL-10	Worse cognitive abilities

### IL-6

IL-6 can be considered a “state marker” of schizophrenia. In this regard, the levels of IL-6 and sIL-6R are elevated in the serum of patients with schizophrenia (40, 41). IL-6 is reported raised in subjects with at-risk mental state (ARMS) and might be a marker of transition from ARMS to schizophrenia (42). IL-6 levels are reported to be high in first-episode psychosis and acutely relapsed patients and to normalize with antipsychotic treatment. An association between treatment-resistant schizophrenia and an elevated level of IL-6 has been described (43) and between the IL-6 level and illness duration (44). Increased IL-6 levels in the CSF of schizophrenia patients in a study by Sasayama et al. further supports the occurrence of inflammatory activity in the CNS in schizophrenia (45). Recently, Arabska et al. (46) reported that serum levels of IL-6 and TNF-α in schizophrenia patients were not significantly different with respect to healthy subjects. Significantly higher plasma levels of IL-6, IL-10, and TNF-α were, however, detected in schizophrenia patients treated with olanzapine or clozapine, as compared with normal controls (47).

### IL-β

Several studies have suggested that IL-1β plays an important role in the etiology and pathophysiology of schizophrenia. Although studies investigating peripheral levels of IL-1β in schizophrenic patients have provided largely inconsistent results, Gilmore et al. proposed the involvement of IL-1β in the possible link between prenatal exposure to infection and schizophrenia (48). An increased release of IL-1β by peripheral monocytes before treatment, and then normalization by antipsychotic medication, has been described in patients with schizophrenia (49). Barak et al. (39) detected significantly lower IL-1β in CSF, other than in serum, of schizophrenic patients compared with controls. In contrast with these results, Söderlund et al. reported that CSF IL-1β concentrations were markedly elevated and hypothesized the activation of the brain immune system in first-episode schizophrenia (38). They suggested that the increase of IL-1β may be normalized or downregulated along the disease course or during prolonged antipsychotic treatment. Thus, whether or not an increase of IL-1β is congenital, acquired during the prodromal phase or absent until the time of first psychotic episode has not yet been clarified. Acute schizophrenics are reported to have significantly elevated IL-1β concentrations in serum, without correlation with age or duration of illness (50). However, serum levels of IL-1β, corrected for age, gender, body mass index, and smoking have been described as no different in patients with schizophrenia and controls and among subtypes of schizophrenia (51). Schizophrenia has been attributed to a dysfunction of brain dopaminergic and glutamatergic circuits, and it is known that IL-1β can induce the conversion of rat mesencephalic progenitor cells into a dopaminergic phenotype (52). In relation to glutamate neurotransmission, the action of IL-1β may include both excitatory and inhibitory components, potentially acting on intercellular brain signaling or altering the expression of genes encoding the enzymes regulating glutamate neurotransmission. However, whether or not the activation of IL-1β is causally related to the development of schizophrenia or is a consequence of associated dopaminergic/glutamatergic dysfunction remains to be established.

### IL-2

Several studies have described altered peripheral levels of IL-2 when compared with healthy controls, as well as a reduction in IL-2 by leukocytes after mitogen stimulation, and such studies also reported that higher levels of IL-2 were indicative of less bad symptomatology and a better cognitive performance. If true, IL-2 may have a key role in the pathophysiology of schizophrenia (53–55).

IL-2R, a key signaling component expressed on T lymphocytes, has been reported to be over-expressed in schizophrenia patients (56), and soluble IL-2R levels are described to be increased in both treatment-naive and treatment-free patients with schizophrenia, as well as in acute and chronic disease patients (57, 58). A study by Bresee and Rapaport (59) suggests that serum-soluble IL-2R levels may represent a biomarker for patients with treatment-resistant psychosis, and a positive correlation between the severity of symptoms and the IL-2R underlines its involvement in schizophrenia.

### IL-8

Other than IL-2 and IL-6, serum levels of IL-8 have also been described as increased in patients with schizophrenia, and correlations between serum IL-2 or IL-8 concentrations at baseline and the therapeutic outcome have been reported (60). Serum levels of IL-8 was statistically significantly elevated in patients with the diagnosis of paranoid schizophrenia, as compared with the control group (61, 62). Such an IL-8 increase may be related to the activation of monocytes and macrophages, as IL-1 and TNF-α released from these cells have been shown to be elevated in schizophrenia. IL-8 expression has also been reported to be significantly increased in the brain tissue of schizophrenia patients, compared with controls, as evaluated by qPCR and immunohistochemistry assay, and confirmed by western blotting. Such elevated levels of IL-8, IL-6, and TNF-α imply that schizophrenia might represent an autoimmune neuropsychiatric spectrum disorder that may emerge during an autoimmune CNS disease (63). Furthermore, there may be connections between the overexpression of IL-8 in patients with schizophrenia and cancer (64). Indeed, an elevation of cytokines may provide insight as to why many patients jointly may have schizophrenia and autoimmune diseases (65, 66).

### IL-3

Genetic studies have shown that the IL-3 and IL-3 receptor alpha subunit (IL-3RA) genes are located near genetic markers associated with schizophrenia (67, 68). With regard to IL-3, studies have reported abnormal levels in chronic schizophrenia patients (69–71). Fu et al. reported that IL-3 levels were significantly decreased in first-episode drug naïve (FEDN) schizophrenic patients, with respect to healthy control subjects and chronic treated schizophrenic patients (72). Additionally, and consistent with previous studies showing significantly higher levels of IL-3 and IL-3-like activity (IL-3-LA) released by peripheral blood mononuclear cells in chronic-medicated patients, IL-3 levels were significantly higher in schizophrenic chronic medicated patients compared with control subjects (70, 71). Such higher IL-3 levels may be associated with disease progression as well as with antipsychotic treatment. A significantly positive association between the Positive and Negative Syndrome Scale (PANSS) general psychopathology subscore and IL-3 was also described in chronic medicated patients with schizophrenia. In contrast, no significant association between IL-3 and any clinical psychopathology in FEDN patients with schizophrenia was observed. As a consequence, it has been speculated that reduced IL-3 levels in FEDN patients might be associated with both neuronal apoptosis and abnormal early development of the CNS, which may potentially be implicated in the pathogenesis of schizophrenia.

### IL-10

Activated macrophages, regulatory T cells, Th2 lymphocytes, and Th3 cells involved in mucosal immunity and protection produce IL-10 (33) that can potentially inhibit the expression of Th1-related cytokines, such as IFN-γ, IL-2, and TNF-α to, thereby, dampen the immune and inflammatory response. Previous studies have found that IL-10 and IL-10 receptors are synthesized in the brain, including by microglia and astrocyte (73). Thus, these may be considered to be an important modulator of the inflammatory response within the CNS (74). Studies performed to understand the relationship between IL-10 and schizophrenia have provided contradictory results. In some studies, an elevation in IL-10 serum levels was detected in patients with schizophrenia (75–78) but other studies failed to replicate this (79, 80). In yet other studies, a decline in serum IL-10 levels was observed in paranoid schizophrenia (62), or in patients with schizophrenia at late stage (81), and no significant differences were detected in IL-10 gene expression levels in peripheral blood cell samples obtained from patients with schizophrenia vs. healthy controls (82). In schizophrenia, elevated peripheral IL-10 levels were associated with the loss of microstructural white matter integrity, supporting the opinion that inflammation is associated with schizophrenia playing a key role in the pathology of microstructural white matter (72).

### IL-12 and IL-23

IL-12 and IL-23 are generated by inflammatory myeloid cells and influence the development of Th1 and Th17 cell responses. IL-12p40 is a component of IL-12 and IL-23 and is induced in excess over the other subunits of IL-12 and IL-23. Plasma levels of IL-12 were investigated in patients with schizophrenia and, likewise, have produced contradictory results (83, 84). A putative role for IL-12p40 as a potential marker in schizophrenia has been proposed, and it has been suggested that its dysregulation may be involved in the pathogenesis of the disorder (85).

### IL-17 and TGF-β

Th17 cells are known to generate the pro-inflammatory cytokine IL-17, which has been implicated across various immune and inflammatory processes (86). Interestingly, recent studies have shown that Th3 and Th17 cells are activated in schizophrenia, and that treatment with antipsychotics mitigated such activation (86, 87). Th3 cells exert their action primarily by secreting transforming growth factor beta-1 (TGF-β1) that plays an immune regulation role by exerting potent anti-inflammatory and immunosuppressive effects to dampen pro-inflammatory cytokine synthesis, natural killer cell activity, and growth of T and B cells. However, TGF-β can, under appropriate conditions, provide proinflammatory functions through its stimulatory effects on inflammatory Th17 cells and, in addition to IL-6, can stimulate IL-17 production. In this regard, it is notable that schizophrenia has been associated with the enhanced expression of TGF-β receptors (88) and the release of TGF-β (87). Data from the literature remains controversial, however. A significant elevation in TGF-β serum levels has been reported in patients with schizophrenia during relapse and first-episode psychosis, as compared with a control group, suggesting that TGF-β may provide a marker for acute exacerbation of schizophrenia (33). However, this has not been confirmed in other studies that could not detect differences between patients with schizophrenia and controls (89). TGF-β signaling has been associated with schizophrenia, as suggested by data from a pathway analysis of a genome wide association study (90). A hyperactivity of TGF-β signaling pathways in schizophrenia may represent a neuroprotective mechanism (91) by promoting the survival of midbrain dopaminergic neurons (92), and increasing neurogenesis within the subventricular zone (93).

A hypothesized role of an IL-17 pathway inbalance in schizophrenia has been strengthened by a study that found an increased activation of Th17 cells in patients with recent onset schizophrenia (94). Borovocanin et al. reported decreased levels of IL-17 in schizophrenia patients (95), and found a significant decrease in Th17 cells. Recently, the percentage of IL-17-producing lymphocytes in peripheral blood of patients with stable schizophrenia and the possible correlation of IL-17 systemic levels with proinflammatory cytokines and cognitive scores was analyzed by Borovocanin et al. (96).

A trend toward a decline in plasma levels of IL-17 was reported by Ding et al. in first-episode schizophrenia patients after 4 weeks of risperidone treatment (86), and Diitrov et al. described a significant reduction in IL-17 levels in chronic schizophrenia patients, as compared with controls (97). In contrast, an increased production of IL-17 following antipsychotic treatment was reported by Himmerich et al. (98). Hence, results on IL-17 in psychosis may be affected by antipsychotic treatment. A recent meta-analysis with drug-naïve first-episode psychosis and healthy control subjects evidenced the absence of significant differences of IL-17 levels between these two groups, suggesting that IL-17 may not be involved in the pathological mechanism underpinning schizophrenia (99) albeit this conclusion derives strictly from the analysis of only drug-naïve FEP patients.

### IL-18

IL-18, a member of the IL-1 family of proinflammatory cytokines, plays an important role in the Th1 response (100) and, hence, its abnormality in schizophrenia would support the activated macrophage theory. In this regard, IL-18 reppresent a link between the immune and nervous systems (101, 102). As IL-18 and its CNS receptors mediate brain neuroinflammation, modulating homeostasis and behavior (102), several studies have been conducted to clarify the involvement of IL-18 in schizophrenia.

Elevated levels of IL-18 were detected in serum of schizophrenic patients by Tanaka et al. (100), possibly as a result of high levels of constitutively or lipopolysaccharide (LPS)-induced IL-18 released by peripheral blood mononuclear cells of schizophrenic patients, as compared with healthy controls (103). Significantly higher IL-18 levels were detected in the serum of chronic schizophrenia patients, with respect to first-episode patients and normal controls, by Xiu et al. (104). Additionally, significant positive associations between IL-18 and the PANSS were observed in chronic patients, whereas no significant association was found between IL-18 and clinical psychopathology in FEP patients. Finally, in chronic patients, there were no differences in IL-18 levels between patients treated with typical or atypical antipsychotics or with dose or duration of treatment (104). The observed elevated IL-18 levels further support the previously described link between autoimmunity and schizophrenia, which also is consistent with the macrophage and T lymphocyte theory.

Genes, such as IL-18, IL-18BP, IL-18R1, IL-18RAP, IL-12B, and IL-12, which mediate IL-18 functions were analyzed by a pathway-oriented approach in schizophrenic patients. Five single nucleotide polymorphisms (SNPs) in four genes were associated with schizophrenia (105). The most prominent among these was rs2272127 at IL-18RAP, which is also associated with herpes simplex virus 1 (HSV1) seropositivity in patients.

Although the IL-18 signaling pathway remains to be fully clarified, its potential role in the pathophysiology of schizophrenia remains speculative rather than proven (106).

### IFN-γ

As IL-18 acts on the immune system—inducing IFN-γ production from Th1 and NK cells—and as IFN-γ is, in turn, the major activating cytokine of macrophages, the involvement of IFN-γ in schizophrenia has also been studied. In this regard, IFN-γ elevations have been frequently noted in patients with schizophrenia and, above all, in first-episode schizophrenia. A correlation has been observed between the spontaneous production of IFN-γ and Positive and Negative Syndrome Scale G subscore. Additionally, a relationship between IFN-γ and the percent whole-brain gray matter suggests that it contributes to the pathophysiology in schizophrenia. Furthermore, a significant reduction of IFN-γ expression in peripheral blood mononuclear cells obtained from schizophrenic patients is in accord with a deficiency in the Th1-dependent immune response as well as with reduced IFN-γ protein levels, as described by Arolt et al. in treated patients (107). Notably, Kim et al. reported that antipsychotic treatment normalized elevated IFN-γ levels (108). Finally, the study by Jemli et al. (109), in part, validates the hypothesis of excessive proinflammatory cytokines in the physiopathology of schizophrenia.

## Cytokine Polymorphisms in Schizophrenia

It has been proposed that a genetic predisposition for schizophrenia has a multifactorial character. While the qualitative and quantitative evaluation of cytokines in biological fluids can provide functional evidence related to disease manifestation, genetic investigations can provide insight into the biological mechanisms underlying susceptibility to disease. Therefore, several studies have been performed to investigate associations between SNPs located within genes encoding cytokines implicated in schizophrenia.

Allelic or genotypic association between the IL-1β C-511T polymorphism and schizophrenia was reported by case-control as well as family studies (110, 111). Other studies have reported no significant association between the IL-1β C-511T or IL-1β C3954T SNP and schizophrenia (112), which disagrees with a meta-analysis in a specific population that reported a moderate association of IL-1β C-511T and C3954T polymorphisms with schizophrenia in Caucasian samples, suggesting that the genetic effect of IL-1β on schizophrenia may be ethnicity dependent (113). The association between the IL-1β GTCC haplotypes G-31A, C-511T, C-1473T, and C-373T and IL-1β C-373T SNP (rs4848306) and schizophrenia in the Polish population was observed by Kapelski et al. (114). Furthermore, schizophrenia in a Japanese population study was significantly associated with IL-1β SNPs (C-373T, C-1473T, and C-511T) (115). Additionally, a study by Sasayama et al. in the Japanese population found an association between the IL-1β A5810G SNP and susceptibility to schizophrenia (116).

Several studies have reported an association between the −174G/C polymorphism in the IL-6 gene and schizophrenia that influences disease risk (117, 118). The study of Paul-Samojedny et al. showed a trend toward a significant difference in genotype distribution and allele frequency between paranoid schizophrenia patients and healthy controls (119), but this was not confirmed in other studies (120–122). The study by Zakharyan et al. demonstrated that the IL-6 −174G/C polymorphism is associated with increased plasma IL-6 in schizophrenia patients and constitutes a risk factor for the disorder (118), and a study by Frydecka et al. indicated that elevated IL-6 in schizophrenia patients is not due to genetic variation (123). However, the IL-6 −174G/C polymorphism may affect the severity of positive symptoms and cognitive impairments observed in schizophrenia and might be the consequence of IL-6 serum levels, thereby, highlighting that schizophrenia is a progressive disorder with low-grade inflammation, essential for its pathophysiology that is then followed by cognitive decline.

The study of Sun et al. reported a signicant association of rs2228145 C allele (Ala allele) of IL-6r with schizophrenia (124). In contrast, Liu et al. found that genetic variants of IL-6 and its receptor are not associated with schizophrenia in Taiwan patients (122). Recently, Kapelski et al. (114) have evaluated the association of functional polymorphisms in several interleukins and interleukin receptor genes and have reported a signicant association of rs2228145 and rs4537545 with schizophrenia. Rafiq et al. (125) found that a common variant of the IL-6r gene increases IL-6r and IL-6 levels, in accord with results of Chan et al. (126) showing that individuals that are homozygous for the minor allele of rs4537545 and its exome SNP exm-rs4537545 had the highest IL-6r levels, as compared with individuals homozygous for the major allele, which had the lowest IL-6r levels. They also reported that SNP, rs7553796 located within the IL-6r gene was associated with increasing levels of the IL-6r protein in blood in homozygous schizophrenia patients.

The association between schizophrenia and IL-8 rs4073, rs2227306, and rs1126647 nucleotide variations was evaluated in a Tunisian population. The rs1126647 polymorphism showed a risk for the development of schizophrenia with a T allele and TT genotype, in particular in the paranoid subtype of schizophrenia. In addition, the rs1126647 SNP appears to predispose more particularly females to the paranoid form. The rare haplotypes TTT, ACT, and TCT haplotypes at rs4073-rs2227306-rs1126647, each comprising the risk allele rs1126647T, were related to the higher risk of paranoid schizophrenia, whereas only the TCT arrangement constitutes a risk factor for general schizophrenia (127).

Sun et al. have reported that IL-10 gene polymorphism is associated with reduced IL-10 production as well as with the susceptibility and the incidence of schizophrenia (128). IL-10 rs1800896, a SNP, is located upstream of the IL-10 gene. The A allele of rs1800896 reduces the production of IL-10, and the known antioxidant actions of IL-10 (129) would therefore be expected to be reduced, which may make long-term treated patients on antipsychotics more vulnerable to the development of tardive dyskinesia (130). Additionally, it has recently been reported that IL-10-592A/C (rs1800872) polymorphisms interact with catechol-o-methyltransferase Val158Met (rs4680) polymorphisms to detrimentally impact cognitive function in schizophrenic patients (131), which is important in the light of cognitive deficits being core symptoms of schizophrenia (132, 133).

The study of Liu et al. (134) evaluated two single promoter polymorphisms, 137 G/C (rs187238) and 607 C/A (rs1946518), of IL-18 in the light of prior studies indicating their potential association with immune-related diseases, including Crohn's disease, diabetes, rheumatoid arthritis, and potentially also Alzheimer's disease, and found that they were not associated with schizophrenia in a Chinese population.

The study of Suchanek-Raif et al. **(author?)** (135) evaluated the potential association between four TNF-α single nucleotide polymorphisms [1031 T/C (rs1799964), 863 C/A (rs1800630), 857 C/T (rs1799724), and 308 G/A (rs1800629)] and schizophrenia in a Caucasian population. Results showed that 1031 T/C, 863 C/A, and 308 G/A are associated with schizophrenia, sex specifically. In fact, high risk of schizophrenia for men is associated with the C/C genotype of 863 G/A SNP. For the −308 G/A SNP, the G/A genotype and A allele were associated with a risk of schizophrenia in women. In contrast, an evaluation of 1031 T/C, 863 C/A, 857 C/T, and 308 G/A SNPs in Caucasian patients with schizophrenia, performed by Tan et al. (136), did not show any significant differences in the SNPs evaluated. Other GWA studies have not found the association between (1031 T/C and 308 G/A) promoter polymorphisms in the TNF-α gene and schizophrenia risk (137). Xiu et al. (138) have suggested an important role for cytokine signaling in mediating the severity of cognitive dysfunction in schizophrenia and reported that the TNF-α 21031T/C polymorphism may not play a role in the predisposition of schizophrenia itself but may be implicated in the cognitive deficits of schizophrenia. In a case-control study, Suchanek-Raif et al. (139) demonstrated that, for the TNFR2 gene, the genetic variants of rs3397, rs1061622, and rs1061624 are linked with a higher risk of developing schizophrenia and a more severe course in men. In contrast, the genotypes with a polymorphic allele for rs3397 SNP appear protective for women. Furthermore, the rs1061624 SNP, in families of people with schizophrenia, might modulate the beginning of the disease process.

Studies aimed at investigating the association between schizophrenia and polymorphisms in genes encoding cytokines shown that the TGF-β1+869T>C gene polymorphism was associated with schizophrenia, especially in females in the context of a TGF-β and estradiol interaction, and the risk of schizophrenia was more than twofold higher in carriers of a T allele (CT+TT genotypes), in comparison with individuals with a CC genotype (140). Also, a significant association between the +874A/T polymorphism of IFN-γ and paranoid schizophrenia has been described, and the minor allele of this polymorphism was correlated with an increased expression of IFN-γ (109). On chromosome 5q, several schizophrenia-related SNPs and haplotypes both in and near the IL-3 cytokine gene have been identified (141). Finally, with the knowledge that T cell networks have consistently been linked to the pathogenesis of schizophrenia, the functional role of the Th17 pathway in schizophrenia was assessed and involved evaluation of the G197A single nucleotide polymorphism associated with production of IL-17A. No association between the IL-17A gene polymorphism and schizophrenia was noted (142).

## Antipsychotic Treatment and Cytokines

Antipsychotic therapy is indispensable in the treatment of schizophrenia. However, phenotypic traits related to drug response, such as responders, delayed responders, or resistant patients, are potentially endophenotypes associated with cytokine profile aberrations. Spreading and/or differentiation of monocytes, T cells and B cells can be modulated by antipsychotic treatment, as well as by a cytokines' and cytokine receptors' gene expression (143, 144). Antipsychotic treatment has been reported to reduce levels of IL-4, IL-6, IL-17, and IL-27 in First Episode Psychosis (FEP) patients (145). In addition, in schizophrenic patients receiving therapy with risperidone, olanzapine, or clozapine, the basal and LPS-induced production of RANTES and IL-18 has reported to be increased (103). A meta-analysis on the effects of antipsychotic treatment evidenced a reduction of select pro- and anti-inflammatory cytokines, such as IL-1β, IL-6, TNF-α, IFN-γ, and TGF-β (33). In a different study, treatment with olanzapine and risperidone reduced serum levels of the anti-inflammatory cytokines IL-1RA and IL-10, while not substantially affecting the expression of seven other evaluated cytokines (146). A study by Song et al. (147) reported that in drug-naïve first-episode schizophrenia, IL-1β levels measured time dependently during 6 months of risperidone treatment decreased initially but then rose gradually over time, and IL-6 and TNF-α levels changed significantly over the treatment course but eventually returned to baseline levels after 6 months. This suggests that risperidone treatment initially provided an anti-inflammatory effect that declined with treatment, possibly due to a drug-induced weight gain side effect. In further studies, still, the levels of anti-inflammatory cytokine IL-10 in peripheral blood were increased by treatment with risperidone (148) and chlorpromazine (149).

*In vitro* studies, likewise, suggest that antipsychotics can potentially modulate the inflammatory response. Haloperidol, a typical antipsychotic, has been reported to normalize increased IL-2 levels (60) and inhibit mitogen-stimulated IL-2 production (150, 151). In LPS-stimulated peripheral blood mononuclear cells, IL-4 and IL-10 levels were increased by haloperidol, and conflicting results have been found in relation to the production of IFN-γ, which in cell cultures may rise (152) or decline—depending on the study conditions (150, 151). Clozapine, risperidone, and quetiapine are common atypical antipsychotics. Quetiapine increased IL-4 levels in LPS-stimulated peripheral blood mononuclear cells, as well as IL-10 production in poly(I:C)-stimulated peripheral blood mononuclear cell cultures—which clozapine and risperidone treatment also significantly increased. All the antipsychotics reduced IFN-γ levels significantly in LPS- and poly(I:C)-stimulated cultures (153). In yet other studies, clozapine has been reported to modulate IL-2, IL-2R, TNF-α and soluble TNF receptors p55 and p75 (154, 155). In schizophrenic patients, risperidone treatment lowered plasma levels of the proinflammatory cytokines TNF-α and IL-6, increased anti-inflammatory cytokine IL-10, and did not impact IL-4 concentrations (156, 157). In closure, a study by Erbaǧci et al. (51) demonstrated that cytokine concentrations in patients responsive and non-responsive to risperidone were not significantly different.

## Cytokine-Based Therapeutic Approaches

In general, the current treatment of schizophrenia involves the use of antipsychotics as the first line of therapy (158). This is often followed by non-steroidal anti-inflammatory drugs (NSAIDS), in addition to vitamins and herbal products targeting the immune system and the inflammatory processes (159). It is known that the inflammatory phenotype should be defined and delineated at the individual level both for research and clinical purposes, in order to evaluate an individual treatment for a patient. A recent meta-analysis study has described the effects of antipsychotics and NSAIDS therapy on cytokine levels, with a reduction in the expression levels of proinflammatory ones, such as IL-18, IL-1β, IL-6, and IL-8 (160, 161).

The wide range of pathological phenotypes underlying schizophrenia and the high possibility of adverse effects during medication supports the need to study new treatment strategies that are currently under investigation. Antibody immunotherapy, tetracycline antibiotics, antirheumatic drugs, neurosteroids, antioxidants, and statins have been reported as possible treatment strategies (159). Hence, in this regard, it is interesting to evaluate the literature as to whether or not they may impact cytokines and cytokine receptors. Clinical trials to evaluate the effects of cytokine modulators have to be careful when considering the pleiotropic and manifold activity of the cytokines (160). Although not yet studied in schizophrenia, some monoclonal antibodies directed toward cytokines have been identified and are either clinically available or in the experimental phase of drug development. In particular, IL-6, TNF-α, and IFN-γ might represent new therapeutic targets.

### IL-6 as a Potential Target

IL-6 is implicated in the development, onset, and progression of several mental disorders and may also be involved in treatment responses. As described by Potvin et al. levels of IL-6 appear to be increased in patients with schizophrenia, both in *in vivo* and *in vitro* models (32). As reported by Behrens et al. (162), it is established that schizophrenic patients have reduced CNS antioxidant defenses, with notable declines in glutathione, a tripeptide that is found in high concentrations in most cells and that is critical to the detoxification of reactive oxygen species and numerous other radicals. Glutathione has been found to be reduced (−27%) in the CSF from drug-naïve patients with schizophrenia, in postmortem studies (−41%) of patients with schizophrenia vs. normal control subjects, and polymorphisms in the genes for key components of glutathione generation [glutamate cysteine ligase modifier subunit (gclm) and the catalytic subunit for glutamate cysteinen ligase (gclc)] are linked to the risk for schizophrenia. Using a subanesthetic ketamine model of schizophrenia in rodents, Behrens et al. demonstrated that IL-6 induces a loss of parvalbumin interneurons that results in a deficiency in inhibitory circuits and the development of a schizophrenic phenotype (162). Other studies have demonstrated that aberrations in parvalbumin interneurons are implicated in schizophrenia and that elevated IL-6 levels in CNS disrupt working memory. Based across this information, clinical trials using a humanized anti-IL-6 receptor monoclonal antibody, Tocilizumab developed and approved for rheumatoid arthritis, were undertaken in five patients with schizophrenia, treated for 4 weeks in an open-label trial. Results showed no significant changes in psychopathology scores, but an improvement in cognition, based on Brief Assessment of Cognition in Schizophrenia (BACS) (163). A more recent randomized, double-blind clinical trial of Tocilizumab was published in 36 clinically stable, moderately symptomatic schizophrenic patients (PANSS >60). The drug was well-tolerated; however, there were no significant effects on positive and negative symptoms and cognitive deficits (164). These somewhat disappointing results may reflect the poor brain penetration of Tocilizumab as well as the schizophrenic patient population evaluated, as greatest elevations in IL-6 levels are generally found in first-episode and acute relapsed subjects, rather than in chronically treated schizophrenia patients—as were evaluated in the Tocilizumab trial.

The anti-IL-6 mAbs, Sirukumab and Siltuximab, have been shown to be effective in reducing depressive symptom severity in patients with rheumatoid arthritis and multicentric Castleman disease (165). In this light, they hold promise in subjects with major depressive disorder and, although their actions on CNS cytokine levels remain to be elucidated, they may be worth evaluating in schizophrenics patients too (166–168).

### TNF-α as a Potential Target

Beyond IL-6, TNF-α is associated with schizophrenia-negative outcome, and thus can be considered a novel drug target for schizophrenia therapy. Antibodies targeting TNF-α, Infliximab, and Golimumab, have been investigated in several clinical trials in patients with major depression. Infliximab activity has been explored by evaluating inflammatory markers (169), sleep parameters (170), and gene expression signatures (171), and these studies supported a depressant response reduction. Bekhbat et al. measured the Infliximab-induced antidepressant response in 26 patients with treatment-resistant depression and demonstrated that subjects with high inflammation and elevated levels of lipids and cholesterols were more responsive to the antidepressant actions of Infliximab. These results support a role of inflammation as a major driver of metabolic dysfunction in patients with depression and provide a framework for the evaluation of TNF-α blocking strategies in schizophrenia (172).

### IFN-γ as a Potential Target

IFN-γ has also been considered a target for cytokine-based immunotherapy in schizophrenic patients. In the light of studies reporting that serum levels of IFN-γ and *in vitro* IFN-γ production after stimulation were lower in samples obtained from unmedicated schizophrenia patients vs. healthy controls (173, 174), a pilot study was undetaken in two treatment-resistant schizophrenia patients in which IFN-γ-1b was administered subcutaneously and psychopathology was assessed weekly with the PANSS (175). In the face of numerous caveats in this small open-label trial, both patients showed a marked clinical improvement, with impressive declines in PANSS total score and good tolerability of the treatment over the relatively short study (6–7 weeks) (175).

### IL-12/IL-23 as Potential Targets

Evaluation of the antidepressant activity of immunomodulatory drugs has also involved investigation of anti-IL-12/IL-23 antibodies. As reported in an interventional clinical trial on psoriatic patients, Ustekinumab treatment (that binds IL-12 and IL-23) for 12 weeks, decreased not only psoriasis symptoms but also depression symptoms, augmenting quality of life (176).

### Anticytokine-Targetted Therapy Synopsis

Collectively, clinical trials with anticytokine therapy have, thus far, been chiefly performed in major depression disorders or in patients afflicted with physical illnesses, such as psoriasis and rheumatoid arthritis (i.e., conditions for which the drugs were primarily developed), and only two studies directly relate to schizophrenic patients, due—in large part—to the ethical limitations of treating patients except as an adjuvant therapy. Results obtained from studies, to date, generally show an improvement in depressive symptoms in the case of adjuvant treatment and with reasonable tolerability, suggesting the possibility of future clinical evaluation in schizophrenic patients. This approach hence represents a promising avenue that warrants further preclinical and clinical research. However, it is necessary to clarify how immunotherapies can best combine with and/or replace available antidepressant treatments, to better define a personalized therapy to reduce side effects and improve the quality of life of patients.

## Conclusion

Cytokines are ubiquitous molecules that act as key messengers for and between immune cells and help to maintain a delicate and intricate balance in the immune system and optimize the continuous cross-talk between the CNS and periphery. In untreated schizophrenia patients, a Th1/Th2 imbalance, with dampened levels of Th1-related cytokines and compensatory elevated Th2-cytokine levels, has been consistently observed—suggesting that the inflammatory process is aberrant and plays a central role in the pathophysiology of schizophrenia. Studies documenting that cytokines are potent rate-limiting signals and that some anticytokine therapies have had promising clinical success open new perspectives on the therapeutic capacity of targeting cytokines/cytokine receptors in neurological disorders. Like other prior promising treatments, cytokine/cytokine receptor-focused therapeutic approaches are not a “magic bullet” capable of improving every individual. Techniques will be required to rationally select out potential responders based on sound scientific principles, which too will require evaluation. In this regard, non-invasive blood sampling, for example, can readily provide the small sample amounts needed to evaluate blood levels of cytokines and represent an attractive way to identify and quantify disease biomarkers. From these same blood samples, extracellular vesicles (also known as exosomes) can be collected and enriched for neuronal and astrocytic origin to provide a window to cytokine/cytokine receptor levels in brain (177–179). Although much research remains to be undertaken, cytokine-based therapies have hugely impacted our understanding and treatment of a broad range of autoimmune disorders and cancers (180) and are among the current top 20 selling drugs worldwide (181). Their pleiotropic actions in brain and potential utility in neurological disorders are only relatively recently being seriously considered. In this regard, identifying the cytokine profiles that precede a psychotic episode could direct strategies for relapse prevention. In addition, biological markers measured during the prodromal phase could have clinical importance in determining diagnosis, further treatment strategies, and prediction of disease progression and treatment response. We have much to gain from better understanding how cytokine imbalances can impact schizophrenia as well as other mental disorders for which current medication is largely inadequate and new treatment strategies are sorely needed.

## Author Contributions

MR and EC designed the work, conducted the literature review, and wrote an initial draft of the article. NG participated in discussions and wrote and revised the manuscript. All authors have revised and approved the final manuscript.

## Conflict of Interest

The authors declare that the research was conducted in the absence of any commercial or financial relationships that could be construed as a potential conflict of interest.

## References

[1] American Psychiatric Association. Diagnostic and Statistical Manual of Mental Disorders. 5th ed. Arlington, VA: American Psychiatric Publishing (2013). 10.1176/appi.books.9780890425596

[2] McDonald C Murray RM. Early and late environmental risk factors for schizophrenia. Brain Res Rev. (2000) 31:130–7. 10.1016/S0165-0173(99)00030-2 10719141

[3] Harrison PJ Weinberger DR. Schizophrenia genes, gene expression, and neuropathology: on the matter of their convergence. Mol. Psychiatry. (2005) 10:40–68. 10.1038/sj.mp.4001558 15263907

[4] Fan X Go DC Henderson DC. Inflammation and schizophrenia. Expert Rev. Neurother. (2007) 7:789–96. 10.1586/14737175.7.7.789 17610386

[5] Khandaker GM Dantzer R Jones PB. Immunopsychiatry: important facts. Psychol. Med. (2017). 47:2229–37. 10.1017/S0033291717000745 28418288 PMC5817424

[6] van Kesteren CFMG Gremmels H de Witte LD Hol EM Van Gool AR Falkai PG. Immune involvement in the pathogenesis of schizophrenia: a meta-analysis on postmortem brain studies. Transl. Psychiatry. (2017) 7:e1075. 10.1038/tp.2017.4 28350400 PMC5404615

[7] Muller N Hofschuster E Ackenheil M Eckstein R. T-cells and psychopathology in schizophrenia: relationship to the outcome of neuroleptic therapy. Acta Psychiatr. Scand. (1993) 87:66–71 10.1111/j.1600-0447.1993.tb03331.x 8093825

[8] Chengappa KN Ganguli R Yang ZW Shurin G Brar JS Rabin RS. Impaired mitogen [PHA] responsiveness and increased autoantibodies in Caucasian schizophrenic patients with the HLA B8/DR3 phenotype. Biol. Psychiatry. (1995) 37:546–9. 10.1016/0006-3223(94)00363-8 7619978

[9] Henneberg AE Horter S Ruffert S. Increased prevalence of antibrain antibodies in the sera from schizophrenic patients. Schizophr. Res. (1994) 14:15e22. 10.1016/0920-9964(94)90004-3 7893617

[10] Momtazmanesh S Zare-Shahabadi A Rezaei N. Cytokine alterations in schizophrenia: an updated review. Front. Psychiatry. (2019) 6:892. 10.3389/fpsyt.2019.00892 31908647 PMC6915198

[11] Garver DL Tamas RL Holcomb JA. Elevated interleukin-6 in the cerebrospinal fluid of a previously delineated schizophrenia subtype. Neuropsychopharmacology. (2003) 28:1515–20. 10.1038/sj.npp.1300217 12799618

[12] Drexhage RC Padmos RC de Wit H Versnel MA Hooijkaas H van der Lely AJ. Patients with schizophrenia show raised serum levels of the pro-inflammatory chemokine CCL2: association with the metabolic syndrome in patients? Schizophr. Res. (2008) 102:352–5. 10.1016/j.schres.2008.03.018 18486454

[13] Padmos RC Hillegers MH Knij EM Vonk R Bouvy A Staal FJ. A discriminating messenger RNA signature for bipolar disorder formed by an aberrant expression of inflammatory genes in monocytes. Arch. Gen. Psychiatry. (2008) 65:395–407. 10.1001/archpsyc.65.4.395 18391128

[14] Wang Y Wei Y Edmiston EK Womer FY Zhang X Duan J. Altered structural connectivity and cytokine levels in schizophrenia and genetic high-risk individuals: associations with disease states and vulnerability. Schizophr. Res. (2020) 223:158–65. 10.1016/j.schres.2020.05.044 32684357

[15] D'Angelo C Reale M Costantini E Di Nicola M Porfilio I de Andrés C. Profiling of canonical and non-traditional cytokine levels in interferon-β-treated relapsing-remitting-multiple sclerosis patients. Front. Immunol. (2018) 4:1240. 10.3389/fimmu.2018.01240 PMC5994428 29915590

[16] Reale M D'Angelo C Costantini E Di Nicola M Yarla NS Kamal MA. Expression profiling of cytokine, cholinergic markers, and amyloid-β deposition in the APPSWE/PS1dE9 mouse model of Alzheimer's disease pathology. J. Alzheimers Dis. (2018) 62:467–76. 10.3233/JAD-170999 29439355 PMC5817902

[17] Reale M Kamal MA Velluto L Gambi D Di Nicola M Greig NH. Relationship between inflammatory mediators, Aβ levels and ApoE genotype in Alzheimer disease. Curr. Alzheimer Res. (2012) 9:447–57. 10.2174/156720512800492549 22272623 PMC5215089

[18] Smith RS Maes M. The macrophage-T-lymphocyte theory of schizophrenia: additional evidence. Med. Hypotheses. (1995) 45:135–41. 10.1016/0306-9877(95)90062-4 8531836

[19] Schwarz MJ Chiang S Müller N Ackenheil M. T-helper-1 and T-helper-2 responses in psychiatric disorders. Brain Behav. Immun. (2001) 15:340–70. 10.1006/brbi.2001.0647 11782103

[20] Wu D Lv P Li F Zhang W Fu G Dai J. Association of peripheral cytokine levels with cerebral structural abnormalities in schizophrenia. Brain Res. (2019) 1724:146463. 10.1016/j.brainres.2019.146463 31526800

[21] Rodrigues-Amorim D Rivera-Baltanás T López M Spuch C Olivares JM Agís-Balboa RC. Schizophrenia: a review of potential biomarkers. J. Psychiatr. Res. (2017) 93:37–49. 10.1016/j.jpsychires.2017.05.009 28578207

[22] Eftekharian MM Omrani MD Arsang-Jang S Taheri M Ghafouri-Fard S. Serum cytokine profile in schizophrenic patients. Hum. Antibodies. (2019) 27:23–29. 10.3233/HAB-180344 30103309

[23] Mosmann TR Coffman RL. TH1 and TH2 cells: different patterns of lymphokine secretion lead to different functional properties. Annu. Rev. Immunol. (1989) 7:145–73. 10.1146/annurev.iy.07.040189.001045 2523712

[24] Harrington LE Hatton RD Mangan PR Turner H Murphy TL Murphy KM. Interleukin 17-producing CD4+ effector T cells develop via a lineage distinct from the T helper type 1 and 2 lineages. Nat. Immunol. (2005) 6:1123–32. 10.1038/ni1254 16200070

[25] Staudt V Bothur E Klein M Lingnau K Reuter S Grebe N. Interferon-regulatory factor 4 is essential for the developmental program of T helper 9 cells. Immunity. (2010) 27:192–202. 10.1016/j.immuni.2010.07.014 20674401

[26] Seder RA Boulay JL Finkelman F Barbier S Ben-Sasson SZ Le Gros G. CD8+ T cells can be primed *in vitro* to produce IL-4. J. Immunol. (1992) 148:1652–6. 1347305

[27] Hauser SL Doolittle TH Lincoln R Brown RH Dinarello CA. Cytokine accumulations in CSF of multiple sclerosis patients: frequent detection of interleukin-1 and tumor necrosis factor but not interleukin-6. Neurology. (1990) 40:1735–9. 10.1212/WNL.40.11.1735 2234430

[28] Reale M De Angelis F Di Nicola M Capello E Di Ioia M De Luca G. Relation between pro-inflammatory cytokines and acetylcholine levels in relapsing-remitting multiple sclerosis patients. Int. J. Mol. Sci. (2012) 13:12656–64. 10.3390/ijms131012656 23202919 PMC3497293

[29] Stanley AC Lacy P. Pathways for cytokine secretion. Physiology. (2010) 25:218–29. 10.1152/physiol.00017.2010 20699468

[30] Lacy P Stow JL. Cytokine release from innate immune cells: association with diverse membrane trafficking pathways. Blood. (2011) 118:9–18. 10.1182/blood-2010-08-265892 21562044

[31] Meyer U Schwarz MJ Müller N. Inflammatory processes in schizophrenia: a promising neuroimmunological target for the treatment of negative/cognitive symptoms and beyond. Pharmacol. Ther. (2011) 132:96–110. 10.1016/j.pharmthera.2011.06.003 21704074

[32] Potvin S Stip E Sepehry AA Gendron A Bah R Kouassi E. Inflammatory cytokine alterations in schizophrenia: a systematic quantitative review. Biol. Psychiatry. (2008) 63:801–8. 10.1016/j.biopsych.2007.09.024 18005941

[33] Miller BJ Buckley P Seabolt W Mellor A Kirkpatrick B. Meta-analysis of cytokine alterations in schizophrenia: clinical status and antipsychotic effects. Biol. Psychiatry. (2011) 70:663–71. 10.1016/j.biopsych.2011.04.013 21641581 PMC4071300

[34] Banks WA Kastin AJ Durham DA. Bidirectional transport of interleukin-1 alpha across the blood-brain barrier. Brain Res. Bull. (1989) 23:433–7. 10.1016/0361-9230(89)90185-8 2611685

[35] Osburg B Peiser C Dömling D Schomburg L Ko YT Voigt K. Effect of endotoxin on expression of TNF receptors and transport of TNF-a at the blood-brain barrier of the rat. Am. J. Physiol. Endocrinol. Metab. (2002) 283:E899–908. 10.1152/ajpendo.00436.2001 12376316

[36] Quagliarello VJ Wispelwey B Long WJ Jr Scheld WM. Recombinant human interleukin-1 induces meningitis and blood-brain barrier injury in the rat. Characterization and comparison with tumor necrosis factor. J. Clin. Invest. (1991) 87:1360. 10.1172/JCI115140 2010549 PMC295174

[37] Buller KM. Role of circumventricular organs in pro-inflammatory cytokine-induced activation of the hypothalamic-pituitary-adrenal axis. Clin. Exp. Pharmacol. Physiol. (2001) 28:581–9. 10.1046/j.1440-1681.2001.03490.x 11458886

[38] Söderlund J Schröder J Nordin C Samuelsson M Walther-Jallow L Karlsson H. Activation of brain interleukin-1b in schizophrenia. Mol. Psychiatry. (2009) 14:1069. 10.1038/mp.2009.52 19920835 PMC2848473

[39] Barak V Barak Y Levine J Nisman B Roisman I. Changes in interleukin-1 beta and soluble interleukin-2 receptor levels in CSF and serum of schizophrenic patients. J. Basic Clin. Physiol. Pharmacol. (1995) 6:61–9. 10.1515/JBCPP.1995.6.1.61 8562579

[40] Chase KA Cone JJ Rosen C Sharma RP. The value of interleukin 6 as a peripheral diagnostic marker in schizophrenia. BMC Psychiatry. (2016) 16:152. 10.1186/s12888-016-0866-x 27206977 PMC4874006

[41] Upthegrove R Manzanares-Teson N Barnes NM. Cytokine function in medication-naive first episode psychosis: a systematic review and meta-analysis. Schizophr. Res. (2014) 155:101–8. 10.1016/j.schres.2014.03.005 24704219

[42] Stojanovic A Martorell L Montalvo I Ortega L Monseny R Vilella E. Increased serum interleukin-6 levels in early stages of psychosis: associations with at-risk mental states and the severity of psychotic symptoms. Psychoneuroendocrinology. (2014) 41:23–32. 10.1016/j.psyneuen.2013.12.005 24495605

[43] Lin A Kenis G Bignotti S Tura GJ De Jong R Bosmans E. The inflammatory response system in treatment-resistant schizophrenia: increased serum interleukin-6. Schizophr. Res. (1998) 32:9–15. 10.1016/S0920-9964(98)00034-6 9690329

[44] Ganguli R Yang Z Shurin G Chengappa KN Brar JS Gubbi AV. (1994). Serum interleukin-6 concentration in schizophrenia: elevation associated with duration of illness. Psychiatry. Res. 51:1–10. 10.1016/0165-1781(94)90042-6 7910974

[45] Sasayama D Hattori K Wakabayashi C Teraishi T Hori H Ota M. Increased cerebrospinal fluid interleukin-6 levels in patients with schizophrenia and those with major depressive disorder. J. Psychiatr. Res. (2013) 47:401–6. 10.1016/j.jpsychires.2012.12.001 23290488

[46] Arabska J Strzelecki D Kozłowska E Brzezińska-Błaszczyk E Wysokiński A. The association between serum levels of TNF-α and IL-6 in schizophrenic patients and their metabolic status - a case control study. J. Neuroimmunol. (2020) 347:577344. 10.1016/j.jneuroim.2020.577344 32777628

[47] Fang X Yu L Wang D Chen Y Wang Y Wu Z. Association between SIRT1, cytokines, and metabolic syndrome in schizophrenia patients with olanzapine or clozapine monotherapy. Front. Psychiatry. (2020) 11:602121. 10.3389/fpsyt.2020.602121 33324265 PMC7723842

[48] Gilmore JH Fredrik Jarskog L Vadlamudi S Lauder JM. Prenatal infection and risk for schizophrenia: IL-1beta, IL-6, and TNFalpha inhibit cortical neuron dendrite development. Neuropsychopharmacology. (2004) 29:1221–9. 10.1038/sj.npp.1300446 15085088

[49] Kowalski J Blada P Kucia K Madej A Herman ZS. Neuroleptics normalize increased release of interleukin- 1 beta and tumor necrosis factor-alpha from monocytes in schizophrenia. Schizophr. Res. (2001) 50:169–75. 10.1016/S0920-9964(00)00156-0 11439237

[50] Katila H Appelberg B Hurme M Rimón R. Plasma levels of interleukin-1 beta and interleukin-6 in schizophrenia, other psychoses, and affective disorders. Schizophr Res. (1994) 12:29–34. 10.1016/0920-9964(94)90081-7 8018583

[51] Erbagci AB Herken H Köylüoglu O Yilmaz N Tarakçioglu M. Serum IL-1b, sIL-2R, IL-6, IL-8 and TNF-a in schizophrenic patients, relation with symptomatology and responsiveness to risperidone treatment. Mediat. Inflamm. (2001) 10:109–15. 10.1080/09629350123895 PMC1781702 11545247

[52] Potter ED Ling ZD Carvey PM. Cytokine-induced conversion of mesencephalic-derived progenitor cells into dopamine neurons. Cell Tissue Res. (1999) 296:235–46. 10.1007/s004410051285 10382268

[53] Mahendran R Chan YH. Interleukin-2 levels in chronic schizophrenia patients. Ann. Acad. Med. Singap. (2004) 33:320–3. 15175772

[54] Ganguli R Rabin BS Belle SH. Decreased interleukin-2 production in schizophrenic patients. Biol. Psychiatry. (1989) 26:427–30. 10.1016/0006-3223(89)90061-9 2788463

[55] Na KS Kim YK. Monocytic, Th1 and th2 cytokine alterations in the pathophysiology of schizophrenia. Neuropsychobiology. (2007) 56:55–63. 10.1159/000111535 18037815

[56] Ghazaryan H Petrek M Boyajyan A. Chronic schizophrenia is associated with over-expression of the interleukin-2 receptor gamma gene. Psychiatry Res. (2014) 217:158–62. 10.1016/j.psychres.2014.03.020 24713359

[57] Rapaport MH McAllister CG Pickar D Nelson DL and Paul SM (1989). Elevated levels of soluble interleukin 2 receptors in schizophrenia. Arch. Gen. Psychiatry. 46:291–2. 10.1001/archpsyc.1989.01810030097017 2784047

[58] Rapaport MH McAllister CG Kim YS Han JH Pickar D Nelson DL. Increased serum soluble interleukin-2 receptors in Caucasian and Korean schizophrenic patients. Biol. Psychiatry. (1994) 35:767–71. 10.1016/0006-3223(94)91137-1 8043705

[59] Bresee C Rapaport MH. Persistently increased serum soluble interleukin-2 receptors in continuously ill patients with schizophrenia. Int. J. Neuropsychopharmacol. (2009) 12:861–5. 10.1017/S1461145709000315 19366488

[60] Zhang XY Zhou DF Cao LY Zhang PY Wu GY Shen YC. Changes in serum interleukin-2,−6, and−8 levels before and during treatment with risperidone and haloperidol: relationship to outcome in schizophrenia. J. Clin. Psychiatry. (2004) 65:940–7. 10.4088/JCP.v65n0710 15291683

[61] Zhang XY Zhou DF Zhang PY Wu GY Cao LY Shen YC. Elevated interleukin 2, interleukin-6 and interleukin-8 serum levels in neuroleptic-free schizophrenia: association with psychopathology. Schizophr. Res. (2002) 57:247–58. 10.1016/S0920-9964(01)00296-1 12223256

[62] Kaminska T Wysocka A Marmurowska-Michalowska H Dubas-Slemp H Kandefer-Szerszen M. Investigation of serum cytokine levels and cytokine production in whole blood cultures of paranoid schizophrenic patients. Arch. Immunol. Ther. Exp. (2001) 49:439–45. 11814238

[63] Yum SY Yum SK Kim T Hwang MY. Clinical perspectives on autoimmune processes in schizophrenia. Psychiatr. Clin. North Am. (2009) 32:795–808. 10.1016/j.psc.2009.09.003 19944884

[64] Xu L Qi X Zhu C Wan L. Activation of IL-8 and its participation in cancer in schizophrenia patients: new evidence for the autoimmune hypothesis of schizophrenia. Neuropsychiatr. Dis. Treat. (2018) 14:3393–403. 10.2147/NDT.S188210 30587991 PMC6298395

[65] Saetre P Emilsson L Axelsson E Kreuger J Lindholm E Jazin E. Inflammation-related genes up-regulated in schizophrenia brains. BMC Psychiatry. (2007) 7:46. 10.1186/1471-244X-7-46 17822540 PMC2080573

[66] Dantzer R O'Connor JC Freund GG Johnson RW Kelley KW. From inflammation to sickness and depression: when the immune system subjugates the brain. Nat. Rev. Neurosci. (2008) 9:46–56. 10.1038/nrn2297 18073775 PMC2919277

[67] Konishi Y Chui DH Kunishita T Yamamura T Higashi Y Tabira T. Demonstration of interleukin-3 receptor-associated antigen in the central nervous system. J. Neurosci. Res. (1995) 41:572–82. 10.1002/jnr.490410503 7563237

[68] Lencz T Morgan TV Athanasiou M Dain B Reed CR Kane JM. Converging evidence for a pseudoautosomal cytokine receptor gene locus in schizophrenia. Mol. Psychiatry. (2007) 12:572–80. 10.1038/sj.mp.4001983 17522711

[69] Bessler H Levental Z Karp L Modai I Djaldetti M Weizman A. Cytokine production in drug-free and neuroleptic-treated schizophrenic patients. Biol. Psychiatry. (1995) 38:297–302. 10.1016/0006-3223(94)00299-I 7495923

[70] Sirota P Schild K Elizur A Djaldetti M Fishman P. Increased interleukin-1 and interleukin-3 like activity in schizophrenic patients. Prog. Neuro Psychopharmacol. Biol. Psychiatry. (1995) 19:75–83. 10.1016/0278-5846(94)00106-R 7708934

[71] Xiu MH Lin CG Tian L Tan YL Chen J Chen S. Increased IL-3 serum levels in chronic patients with schizophrenia: associated with psychopathology. Psychiatry Res. (2015) 229:225–9. 10.1016/j.psychres.2015.07.029 26208986

[72] Fu YY Zhang T Xiu MH Tang W Han M Yun LT. Altered serum levels of interleukin-3 in first-episode drug-naive and chronic medicated schizophrenia. Schizophr Res. (2016) 176:196–200. 10.1016/j.schres.2016.05.010 27237600

[73] Ledeboer A Jekich BM Sloane EM Mahoney JH Langer SJ Milligan ED. Intrathecal interleukin-10 gene therapy attenuates paclitaxel-induced mechanical allodynia and proinflammatory cytokine expression in dorsal root ganglia in rats. Brain Behav. Immun. (2007) 21:686–98. 10.1016/j.bbi.2006.10.012 17174526 PMC2063454

[74] Strle K Zhou JH Shen WH Broussard SR Johnson RW Freund GG. Interleukin-10 in the brain. Crit. Rev. Immunol. (2001) 21:427–49. 10.1615/CritRevImmunol.v21.i5.20 11942558

[75] Maes M Bocchio Chiavetto L Bignotti S Battisa Tura GJ Pioli R Boin F. Increased serum interleukin-8 and interleukin-10 in schizophrenic patients resistant to treatment with neuroleptics and the stimulatory effects of clozapine on serum leukemia inhibitory factor receptor. Schizophr. Res. (2002) 54:281–91. 10.1016/S0920-9964(00)00094-3 11950553

[76] Kunz M Ceresér KM Goi PD Fries GR Teixeira AL Fernandes BS. Serum levels of IL-6, IL-10 and TNF-α in patients with bipolar disorder and schizophrenia: differences in pro- and anti-inflammatory balance. Braz. J. Psychiatry. (2011) 33:268–74. 10.1590/S1516-44462011000300010 21971780

[77] Rothermundt M Arolt V Weitzsch C Eckhoff D Kirchner H. Production of cytokines in acute schizophrenic psychosis. Biol. Psychiatry. (1996) 40:1294–7. 10.1016/S0006-3223(96)00360-5 8959295

[78] Cazzullo CL Scarone S Grassi B Vismara C Trabattoni D Clerici M. Cytokines production in chronic schizophrenia patients with or without paranoid behaviour. Prog. Neuropsychopharmacol. Biol. Psychiatry. (1998) 22:947–57. 10.1016/S0278-5846(98)00059-1 9789879

[79] O'Brien SM Scully P Dinan TG. Increased tumor necrosis factor-alpha concentrations with interleukin-4 concentrations in exacerbations of schizophrenia. Psychiatry Res. (2008) 160:256–62. 10.1016/j.psychres.2007.11.014 18722671

[80] Kubistova A Horacek J Novak T. Increased interleukin-6 and tumor necrosis factor alpha in first episode schizophrenia patients versus healthy controls. Psychiatr. Danub. (2012) 1:S153-6. 22945211

[81] Pedrini M Massuda R Fries GR de Bittencourt Pasquali MA Schnorr CE. Similarities in serum oxidative stress markers and inflammatory cytokines in patients with overt schizophrenia at early and late stages of chronicity. J. Psychiatr. Res. (2012) 46:819–24. 10.1016/j.jpsychires.2012.03.019 22520512

[82] Freudenreich O Brockman MA Henderson DC Evins AE Fan X Walsh JP. Analysis of peripheral immune activation in schizophrenia using quantitative reverse-transcription polymerase chain reaction (RT-PCR). Psychiatry Res. (2010) 176:99–102. 10.1016/j.psychres.2008.11.007 20132993 PMC2844464

[83] Kim YK Suh IB Kim H Han CS Lim CS Choi SH. The plasma levels of interleukin-12 in schizophrenia, major depression, and bipolar mania: effects of psychotropic drugs. Mol. Psychiatry. (2002) 7:1107–14. 10.1038/sj.mp.4001084 12476326

[84] Ozbey U Tug E Kara M Namli M. The value of interleukin-12B (p40) gene promoter polymorphism in patients with schizophrenia in a regionof East Turkey. Psychiatry Clin. Neurosci. (2008) 62:307–12. 10.1111/j.1440-1819.2008.01798.x 18588591

[85] Bedrossian N Haidar M Fares J Kobeissy FH Fares Y. Inflammation and elevation of Interleukin-12p40 in patients with schizophrenia. Front. Mol. Neurosci. (2016) 9:16. 10.3389/fnmol.2016.00016 27047333 PMC4801873

[86] Ding M Song X Zhao J Gao J Li X Yang G. Activation of Th17 cells in drug naive, first episode schizophrenia. Prog. Neuropsychopharmacol. Biol. Psychiatry. (2014) 51:78–82. 10.1016/j.pnpbp.2014.01.001 24447943

[87] Kim YK Myint AM Lee BH Han CS Lee HJ Kim DJ. Th1, Th2 and Th3 cytokine alteration in schizophrenia. Prog. Neuropsychopharmacol. Biol. Psychiatry. (2004) 28:1129–34. 10.1016/j.pnpbp.2004.05.047 15610925

[88] Numata S Ueno S Iga J Yamauchi K Hongwei S Hashimoto R. TGFBR2 gene expression and genetic association with schizophrenia. J. Psychiatr. Res. (2008) 42:425–32. 10.1016/j.jpsychires.2007.04.002 17560608

[89] El Kissi Y Samoud S Mtiraoui A Letaief L Hannachi N Ayachi M. Increased Interleukin-17 and decreased BAFF serum levels in drug-free acute schizophrenia. Psychiatry Res. (2015) 225:58–63. 10.1016/j.psychres.2014.10.007 25453636

[90] Jia P Wang L Meltzer HY Zhao Z. Common variants conferring risk of schizophrenia: a pathway analysis of GWAS data. Schizophr. Res. (2010) 122:38–42. 10.1016/j.schres.2010.07.001 20659789 PMC2933424

[91] Dobolyi A Vincze C Pal G Lovas G. The neuroprotective functions of transforming growth factor beta proteins. Int. J. Mol. Sci. (2012) 13:8219–58. 10.3390/ijms13078219 22942700 PMC3430231

[92] Krieglstein K Suter-Crazzolara C Fischer WH Unsicker K. TGF-beta superfamily members promote survival of midbrain dopaminergic neurons and protect them against MPP+ toxicity. EMBO J. (1995) 14:736–42. 10.1002/j.1460-2075.1995.tb07052.x 7882977 PMC398139

[93] Mathieu P Piantanida AP Pitossi F. Chronic expression of transforming growth factor-beta enhances adult neurogenesis. Neuroimmunomodulation. (2010) 17:200–1. 10.1159/000258723 20134202

[94] Drexhage RC Hoogenboezem TA Cohen D Versnel MA Nolen WA van Beveren NJM. An activated set point of T-cell and monocyte inflammatory networks in recent-onset schizophrenia patients involves both pro- and anti-inflammatory forces. Int. J. Neuropsychopharmacol. (2011) 14:746–55. 10.1017/S1461145710001653 21255481

[95] Borovocanin M Jovanovic I Radosavijevic G Djukic Dejanovic S Bankovic D Arsenijevic N. Elevated serum levels of type-2 cytokine and low IL-17 in first episode psychosis and schizophrenia in relapse. J. Psychiatr. Res. 46:1421–6. 10.1016/j.jpsychires.2012.08.016 22974591

[96] Borovcanin MM Minic Janicijevic S Jovanovic IP Gajovic NM Jurisevic MM Arsenijevic NN. Type 17 immune response facilitates progression of inflammation and correlates with cognition in stable schizophrenia. Diagnostics. (2020) 10:926. 10.3390/diagnostics10110926 33182582 PMC7698203

[97] Dimitrov DH Lee S Yantis J Valdez C Paredes RM Braida N. Differential correlations between inflammatory cytokines and psychopathology in veterans with schizophrenia: potential role for IL-17 pathway. Schizophr. Res. (2013) 151:29–35. 10.1016/j.schres.2013.10.019 24210870

[98] Himmerich H SchoÅNnherr J Fulda S Sheldrick AJ Bauer K Sack U. Impact of antipsychotics on cytokine production *in-vitro*. J. Psychiatr. Res. (2011) 45:1358–65. 10.1016/j.jpsychires.2011.04.009 21592521

[99] Fang X Zhang Y Fan W Tang W Zhang C. Interleukin-17 alteration in first-episode psychosis: a meta-analysis. Mol. Neuropsychiatry. (2017) 3:135–40. 10.1159/000481661 29594132 PMC5836254

[100] Tanaka KF Shintani F Fujii Y Yagi G Asai M. Serum interleukin-18 levels are elevated in schizophrenia. Psychiatry Res. (2000) 96:75e80. 10.1016/S0165-1781(00)00196-7 10980328

[101] Felderhoff-Mueser U Schmidt OI Oberholzer A Bührer C Stahel PF. IL-18: a key player in neuroinflammation and neurodegeneration? Trends Neurosci. (2005) 28:487–93. 10.1016/j.tins.2005.06.008 16023742

[102] Alboni S Cervia D Sugama S Conti B. Interleukin 18 in the CNS. J. Neuroinflammation. (2010) 7:9. 10.1186/1742-2094-7-9 20113500 PMC2830964

[103] Reale M Patruno A De Lutiis MA Pesce M Felaco M Di Giannantonio M. Dysregulation of chemo-cytokine production in schizophrenic patients versus healthy controls. BMC Neurosci. (2011) 12:13. 10.1186/1471-2202-12-13 21266029 PMC3038147

[104] Xiu MH Chen DC Wang D Zhang K Dong A Tang W. Elevated interleukin-18 serum levels in chronic schizophrenia: association with psychopathology. J. Psychiatr. Res. (2012) 46:1093–8. 10.1016/j.jpsychires.2012.04.026 22647522

[105] Shirts BH Wood J Yolken RH Nimgaonkar VL. Comprehensive evaluation of positional candidates in the IL-18 pathway reveals suggestive associations with schizophrenia and herpes virus seropositivity. Am. J. Med. Genet. Part B. (2008) 147:343e350. 10.1002/ajmg.b.30603 18092318

[106] Syed AAS He L Shi Y Mahmood S. Elevated levels of IL-18 associated with schizophrenia and first episode psychosis: a systematic review and meta-analysis. Early Interv. Psychiatry. (2020). 10.1111/eip.13031. [Epub ahead of print]. 32902142

[107] Arolt V Rothermundt M Wandinger KP Kirchner H. (2000). Decreased *in vitro* production of interferon-gamma and interleukin-2 in whole blood of patients with schizophrenia during treatment. Mol. Psychiatry. 5:150–8. 10.1038/sj.mp.4000650 10822342

[108] Kim YK Lee MS Suh KY. Decreased interleukin-2 production in Korean schizophrenic patients. Biol. Psychiatry. (1998) 43:701–4. 10.1016/S0006-3223(97)00357-0 9583005

[109] Jemli A Eshili A Trifa F Mechri A Zaafrane F Gaha L. Association of the IFN-γ (+874A/T) genetic polymorphism with paranoid schizophrenia in tunisian population. Immunol. Invest. (2017) 46:159–71. 10.1080/08820139.2016.1237523 27819519

[110] Laurent C Thibaut F Ravassard P Campion D Samolyk D Lafargue C. Detection of two new polymorphic sites in the human interleukin-1 beta gene: lack of association with schizophrenia in a French population. Psychiatr. Genet. (1997) 7:103–5. 10.1097/00041444-199723000-00002 9323321

[111] Shibuya M Watanabe Y Nunokawa A Egawa J Kaneko N Igeta H. Interleukin 1 beta gene and risk of schizophrenia: detailed case- control and family-based studies and an updated meta-analysis. Hum. Psychopharmacol. (2014) 29:31–7. 10.1002/hup.2365 24155145

[112] Shirts BH Wood J Yolken RH Nimgaonkar VL. Association study of IL10, IL1beta, and IL1RN and schizophrenia using tag SNPs from a comprehensive database: suggestive association with rs16944 at IL1beta. Schizophr. Res. (2006) 88:235–44. 10.1016/j.schres.2006.06.037 16905295

[113] Xu M He L. Convergent evidence shows a positive association of interleukin- 1 gene complex locus with susceptibility to schizophrenia in the Caucasian population. Schizophr. Res. (2010) 120:131–42. 10.1016/j.schres.2010.02.1031 20347268

[114] Kapelski P Skibinska M Maciukiewicz M Wilkosc M Frydecka D Groszewska A. Association study of functional polymorphisms in interleukins and interleukin receptors genes: IL1A, IL1B, IL1RN, IL6, IL6R, IL10, IL10RA and TGFB1 in schizophrenia in Polish population, *Schizophr*. Res. (2015) 169:1–9. 10.1016/j.schres.2015.10.008 26481614

[115] Yoshida M Shiroiwa K Mouri K Ishiguro H Supriyanto I Ratta-Apha W. Haplotypes in the expression quantitative trait locus of interleukin-1beta gene are associated with schizophrenia. Schizophr. Res. (2012) 140:185–91. 10.1016/j.schres.2012.06.031 22804923

[116] Sasayama D Hori H Teraishi T Hattori K Ota M Iijima Y. Possible association between interleukin-1beta gene and schizophrenia in a Japanese population. Behav. Brain Funct. 7:35. 10.1186/1744-9081-7-35 21843369 PMC3168401

[117] Terry CF Loukaci V Green FR. Cooperative influence of genetic polymorphisms on interleukin 6 transcriptional regulation. J. Biol. Chem. (2000) 275:18138–44. 10.1074/jbc.M000379200 10747905

[118] Zakharyan R Petrek M Arakelyan A Mrazek F Atshemyan S Boyajyan A. Interleukin-6 promoter polymorphism and plasma levels in patients with schizophrenia. Tissue Antigens. (2012) 80:136–42. 10.1111/j.1399-0039.2012.01886.x 22571276

[119] Paul-Samojedny M Kowalczyk M Suchanek R Owczarek A Fila-Danilow A Szczygiel A. Functional polymorphism in the interleukin-6 and interleukin-10 genes in patients with paranoid schizophrenia: a case–control study. J. Mol. Neurosci. (2010) 42:112–9. 10.1007/s12031-010-9365-6 20393813

[120] Paul-Samojedny M Owczarek A Kowalczyk M Suchanek R Palacz M Kucia K. Association of interleukin 2 (IL-2), interleukin 6 (IL-6), and TNF-alpha (TNFalpha) gene polymorphisms with paranoid schizophrenia in a Polish population. J. Neuropsychiatry Clin. Neurosci. (2013) 25:72–82. 10.1176/appi.neuropsych.12020021 23487197

[121] Debnath M Mitra B Bera NK Chaudhuri TK Zhang YP. Lack of association of IL-6 (-174 G>C) and TNF-alpha (-238 G>A) variants with paranoid schizophrenia in Indian Bengalee population. Cytokine. (2013) 61:455–8. 10.1016/j.cyto.2012.10.028 23265967

[122] Liu YL Liu CM Fann CS Yang WC Chen YH Tseng LJ. Genetic variants of IL-6 and its receptor are not associated with schizophrenia in Taiwan. Neurosci. Lett. (2010) 468:330–3. 10.1016/j.neulet.2009.11.026 19914334

[123] Frydecka D Misiak B Pawlak-Adamska E Karabon L Tomkiewicz A Sedlaczek P. Interleukin-6: the missing element of the neurocognitive deterioration in schizophrenia? The focus on genetic underpinnings, cognitive impairment and clinical manifestation. Eur. Arch. Psychiatry Clin. Neurosci. (2015) 265:449–59. 10.1007/s00406-014-0533-5 25214388 PMC4540774

[124] Sun S Wang F Wei J Cao LY Qi LY Xiu MH. Association between interleukin-6 receptor polymorphism and patients with schizophrenia. Schizophr. Res. (2008) 102:346–7. 10.1016/j.schres.2008.04.018 18508242

[125] Rafiq S Frayling TM Murray A Hurst A Stevens K Weedon MN. A common variant of the interleukin 6 receptor (IL-6r) gene increases IL-6r and IL-6 levels, without other inflammatory effects. Genes Immunity. (2007) 8:552–9. 10.1038/sj.gene.6364414 17671508 PMC2668154

[126] Chan MK Cooper JD Heilmann-Heimbach S Frank J Witt SH Nöthen MM. Associations between SNPs and immune-related circulating proteins in schizophrenia. Sci. Rep. (2017) 7:12586. 10.1038/s41598-017-12986-0 28974776 PMC5626704

[127] Ben Afia A Aflouk Y Saoud H Zaafrane F Gaha L Bel Hadj Jrad B. Inteurleukin-8 gene variations and the susceptibility to schizophrenia. Psychiatry Res. (2020) 293:113421. 10.1016/j.psychres.2020.113421 32920525

[128] Sun H Wang F Fan H Yan Q Cui K Yuan W. Interaction of polymorphisms of IL10 and DBH was associated with general symptoms of PANSS with TD in Chinese Han schizophrenic patients. PLoS ONE. (2013) 8:e70963 10.1371/journal.pone.0070963 23951054 PMC3737228

[129] Haddad JJ Fahlman CS. Redox- and oxidant-mediated regulation of interleukin-10: an anti-inflammatory, antioxidant cytokine? Biochem. Biophys. Res. Commun. (2002) 297:163–76. 10.1016/S0006-291X(02)02094-6 12237098

[130] Choi K-Y Choo JM Lee Y-J Lee Y Cho C-H Kim S-H. Association between the IL10 rs1800896 polymorphism and tardive dyskinesia in schizophrenia. Psychiatry Investig. (2020) 17:1031–6. 10.30773/pi.2020.0191 33059393 PMC7596282

[131] Wang J Xu H Wang D Wei G Zhou H Wang L. The interactive effect of genetic polymorphisms of IL-10 and COMT on cognitive function in schizophrenia. J. Psychiatr. Res. (2020). 10.1016/j.jpsychires.2020.10.021. [Epub ahead of print]. 33127070

[132] Barch DM Ceaser A. Cognition in schizophrenia: core psychological and neural mechanisms. Trends Cognit. Sci. (2012) 16:27–34. 10.1016/j.tics.2011.11.015 22169777 PMC3860986

[133] Foussias G Remington G. Negative symptoms in schizophrenia: avolition and Occam's razor. Schizophr. Bull. (2010) 36, 359–69. 10.1093/schbul/sbn094 18644851 PMC2833114

[134] Liu J Liu J Zhou Y Li S Li Y Song X. (2011). Association between promoter variants of interleukin-18 and schizophrenia in a Han Chinese population. DNA Cell Biol. 21:456–61. 10.1089/dna.2011.1221 21510800

[135] Suchanek-Raif R Raif P Kowalczyk M Paul-Samojedny M Kucia K Merk W. Promoter polymorphisms of TNF-α gene as a risk factor for schizophrenia. Arch. Med. Res. (2018) 49:248–54. 10.1016/j.arcmed.2018.09.007 30268704

[136] Tan E-C Chong S-A Tan C-H Teo Y-Y Peng K Mahendran R. Tumor necrosis factor-alpha gene promoter polymorphisms in chronic schizophrenia. Biol. Psychiatry. (2003) 54:1205–11. 10.1016/S0006-3223(03)00345-7 14643088

[137] Cross-Disorder Group of the Psychiatric Genomics Consortium. Identification of risk loci with shared effects on five major psychiatric disorders: a genome-wide analysis. Lancet. (2013) 381:1371–9. 10.1016/S0140-6736(12)62129-1 23453885 PMC3714010

[138] Xiu MH Man LJ Wang D Du X Yin G Zhang Y. Tumor necrosis factor-alpha−1031T/C polymorphism is associated with cognitive deficits in chronic schizophrenia patients versus healthy controls. Am. J. Med. Genet. B Neuropsychiatr. Genet. (2018) 177:379–87. 10.1002/ajmg.b.32622 29633506

[139] Suchanek-Raif R Raif P Kowalczyk M Paul-Samojedny M Kucia K Merk W. Polymorphic Variants of *TNFR2* gene in schizophrenia and its interaction with−308G/A *TNF-*α gene polymorphism. Mediat. Inflamm. (2018) 2018:8741249. 10.1155/2018/8741249 30254506 PMC6142735

[140] Frydecka D Misiak B Beszlej JA Karabon L Pawlak-Adamska E Tomkiewicz A. Genetic variants in transforming growth factor-b gene [TGFB1] affect susceptibility to schizophrenia. Mol. Biol. Rep. (2013) 40:5607–14. 10.1007/s11033-013-2662-8 24065520 PMC3824289

[141] Chen X Wang X Hossain S O'Neill FA Walsh D van den Oord E. Interleukin 3 and schizophrenia: the impact of sex and family history. Mol. Psychiatry. (2007) 12:273–82. 10.1038/sj.mp.4001932 17179997

[142] Subbanna M Shivakumar V Talukdar PM Narayanaswamy JC Venugopal D Berk M. Role of IL-6/RORC/IL-22 axis in driving Th17 pathway mediated immunopathogenesis of schizophrenia. Cytokine. (2018) 111:112–8. 10.1016/j.cyto.2018.08.016 30138899

[143] Chen M-L Wu S Tsai T-C Wang L-K Tsai F-M. Regulation of macrophage immune responses by antipsychotic drugs. Immunopharmacol. Immunotoxicol. (2013) 35:573–80. 10.3109/08923973.2013.828744 23981042

[144] Debnath M. Adaptive immunity in schizophrenia: functional implications of T cells in the etiology, course and treatment. J. Neuroimmune. Pharmacol. (2015) 10:610–9. 10.1007/s11481-015-9626-9 26162591

[145] Borovocanin M Jovanovic I Radosavljevic G Djukic Dejanovic S Stefanovic V Arsenijevic N. (2013). Antipsychotics can modulate the cytokine profile in schizophrenia: attenuation of the type-2 inflammatory response. Schizophr. Res. 147:103–9. 10.1016/j.schres.2013.03.027 23602340

[146] De Witte L Tomasik J Schwarz E Guest PC Rahmoune H Kahn RS. Cytokine alterations in first-episode schizophrenia patients before and after antipsychotic treatment. Schizophr. Res. (2014) 154:23–9. 10.1016/j.schres.2014.02.005 24582037

[147] Song X Fan X Li X Zhang W Gao J Zhao J. Changes in pro-inflammatory cytokines and body weight during 6-month risperidone treatment in drug naïve, first-episode schizophrenia. Psychopharmacology. (2014) 231:319–25. 10.1007/s00213-013-3382-4 24337064

[148] Cazzullo CL Sacchetti E Galluzzo A Panariello A Adorni A Pegoraro M. Cytokine profiles in schizophrenic patients treated with risperidone: a 3-month follow-up study. Prog. Neuropsychopharmacol. Biol. Psychiatry. (2002) 26:33–9. 10.1016/S0278-5846(01)00221-4 11853116

[149] Tarazona R Gonzalez-Garcia A Zamzami N Marchetti P Frechin N Gonzalo JA. Chlorpromazine amplifies macrophage-dependent IL-10 production *in vivo*. J. Immunol. (1995) 154:861–70. 7814889

[150] Leykin I Mayer R Shinitzky M. Short and long-term immunosuppressive effects of clozapine and haloperidol. Immunopharmacology. (1997) 37:75–86. 10.1016/S0162-3109(97)00037-4 9285246

[151] Szuster-Ciesielska A Słotwiska M Stachura A Marmurowska-Michałowska H Kandefer-Szersze M. Neuroleptics modulate cytokine and reactive oxygen species production in blood leukocytes of healthy volunteers. Arch. Immunol. Ther. Exp. (2004) 52:59–67. 15053234

[152] Rudolf S Peters M Rothermundt M Arolt V Kirchner H. The influence of typical and atypical neuroleptic drugs in the production of interleukin-2 and interferon-gamma *in vitro*. Neuropsychobiology. (2002) 46:180–5. 10.1159/000067807 12566934

[153] Al-Amin M Nasir U Mahmud Reza H. Effects of antipsychotics on the inflammatory response system of patients with schizophrenia in peripheral blood mononuclear cell cultures. Clin. Psychopharmacol. Neurosci. (2013) 11:144–51. 10.9758/cpn.2013.11.3.144 24465251 PMC3897763

[154] Pollmächer T Hinze-Selch D Mullington J. Effects of clozapine on plasma cytokine and soluble cytokine receptor levels. J. Clin. Psychopharmacol. (1996) 16:403–9. 10.1097/00004714-199610000-00011 8889915

[155] Huanzhong L Cai H Ren Z Zhong J Li J. Clozapine regulates cytokines, T-cell subsets and immunoglobulins serum levels in MK-801-evoked schizophrenia rat. Int. J. Pharmacol. (2015) 11:596–603. 10.3923/ijp.2015.596.603

[156] Ajami A Abedian F Hamzeh-Hosseini S Akbarian E Alizadeh-Navaei R Taghipour M. Serum TNF-α, IL-10 and IL-2 in schizophrenic patients before and after treatment with risperidone and clozapine. Iran J Immunol. (2014) 11:200–9. 25265997 10.22034/iji.2014.16780

[157] Noto C Ota VK Gouvea ES Rizzo LB Spindola LM Honda PH. Effects of risperidone on cytokine profile in drug-naïve first-episode psychosis. Int. J. Neuropsychopharmacol. (2014) 18:pyu042. 10.1093/ijnp/pyu042 25522386 PMC4360233

[158] Patel KR Cherian J Gohil K Atkinson D. Schizophrenia: overview and treatment options. PT. (2014) 39:638–45. PMC4159061 25210417

[159] Melbourne JK Feiner B Rosen C Sharma RP. Targeting the immune system with pharmacotherapy in schizophrenia. Curr. Treat Options Psychiatry. (2017) 4:139–51. 10.1007/s40501-017-0114-0 28674674 PMC5493152

[160] Kroken RA Sommer IE Steen VM Dieset I Johnsen E. Constructing the immune signature of schizophrenia for clinical use and research; an integrative review translating descriptives into diagnostics. Front. Psychiatry. (2019) 9:753. 10.3389/fpsyt.2018.00753 30766494 PMC6365449

[161] Sommer IE van Westrhenen R Begemann MJH de Witte LD Leucht S Kahn RS. Efficacy of anti-inflammatory agents to improve symptoms in patients with schizophrenia: an update. Schizophr. Bull. (2014) 40:181–91. 10.1093/schbul/sbt139 24106335 PMC3885306

[162] Behrens MM Ali SS Dugan LL. Interleukin-6 mediates the increase in NADPH-oxidase in the ketamine model of schizophrenia. J. Neurosci. (2008) 28:13957–66. 10.1523/JNEUROSCI.4457-08.2008 19091984 PMC2752712

[163] Miller BJ Dias JK Lemos HP Buckley PF. An open-label, pilot trial of adjunctive tocilizumab in schizophrenia. J. Clin. Psychiatry. (2016) 77:275–6. 10.4088/JCP.15l09920 26930525

[164] Girgis RR Ciarleglio A Choo T Haynes G Bathon JM Cremers S. A randomized, double-blind, placebo-controlled clinical trial of tocilizumab, an interleukin-6 receptor antibody, for residual symptoms in schizophrenia. Neuropsychopharmacology. (2018) 43:1317–23. 10.1038/npp.2017.258 29090685 PMC5916349

[165] Sun Y Wang D Salvadore G Hsu B Curran M Casper C. The effects of interleukin-6 neutralizing antibodies on symptoms of depressed mood and anhedonia in patients with rheumatoid arthritis and multicentric Castleman's disease. Brain Behav Immun. (2017) 66:156–64. 10.1016/j.bbi.2017.06.014 28676350

[166] Szepietowski C Nilganuwong S Wozniacka A Kuhn A Nyberg F van Vollenhoven RF. Phase I, randomized, double-blind, placebo-controlled, multiple intravenous, dose-ascending study of sirukumab in cutaneous or systemic lupus erythematosus. Arthritis Rheum. (2013) 65:2661–71. 10.1002/art.38091 23896980

[167] Zhou AJ Lee Y Salvadore G Hsu B Fonseka TM Kennedy SH. Sirukumab: a potential treatment for mood disorders? Adv. Ther. (2017) 34:78–90. 10.1007/s12325-016-0455-x 27913990 PMC5216059

[168] Wittenberg GM Stylianou A Zhang Y Sun Y Gupta A Jagannatha PS. Effects of immunomodulatory drugs on depressive symptoms: a mega-analysis of randomized, placebo-controlled clinical trials in inflammatory disorders. Mol Psychiatry. (2020) 25:1275–85. 10.1038/s41380-019-0471-8 31427751 PMC7244402

[169] Raison CL Rutherford RE Woolwine BJ Shuo C Schettler P Drake DF. A randomized controlled trial of the tumor necrosis factor antagonist infliximab for treatment-resistant depression: the role of baseline inflammatory biomarkers. JAMA Psychiatry. (2013) 70:31–41. 10.1001/2013.jamapsychiatry.4 22945416 PMC4015348

[170] Weinberger JF Raison CL Rye DB Montague AR Woolwine BJ Felger JC. Inhibition of tumor necrosis factor improves sleep continuity in patients with treatment resistant depression and high inflammation. Brain Behav. Immun. (2015) 47:193–200. 10.1016/j.bbi.2014.12.016 25529904 PMC4468009

[171] Mehta D Raison CL Woolwine BJ Haroon E Binder EB Miller AH. Transcriptional signatures related to glucose and lipid metabolism predict treatment response to the tumor necrosis factor antagonist infliximab in patients with treatment-resistant depression. Brain Behav. Immun. (2013) 31:205–15. 10.1016/j.bbi.2013.04.004 23624296 PMC3673885

[172] Bekhbat M Chu K Le NA Woolwine BJ Haroon E Miller AH. Glucose and lipid-related biomarkers and the antidepressant response to infliximab in patients with treatment-resistant depression. Psychoneuroendocrinology. (2018) 98:222–9. 10.1016/j.psyneuen.2018.09.004 30249443 PMC6214671

[173] Rothermundt M Arolt V Leadbeater J Peters M Rudolf S Kirchner H. Cytokine production in unmedicated and treated schizophrenic patients. Neuroreport. (2000) 11:3385–8. 11059907 10.1097/00001756-200010200-00024

[174] Schwarz MJ Müller N Riedel M Ackenheil M. The Th2-hypothesis of schizophrenia: a strategy to identify a subgroup of schizophrenia caused by immune mechanisms. Med. Hypotheses. (2001) 56:483–6. 10.1054/mehy.2000.1203 11339852

[175] Grüber L Bunse T Weidinger E Reichard H Müller N. Adjunctive recombinant human interferon gamma-1b for treatment-resistant schizophrenia in 2 patients. J. Clin. Psychiatry. (2014) 75:1266–7. 10.4088/JCP.14l09005 25470089

[176] Langley RG Feldman SR Han C Schenkel B Szapary P Hsu M-C. Ustekinumab significantly improves symptoms of anxiety, depression, and skin-related quality of life in patients with moderate-to-severe psoriasis: results from a randomized, double- blind, placebo-controlled phase III trial. J. Am. Acad. Dermatol. (2010) 63:457–65. 10.1016/j.jaad.2009.09.014 20462664

[177] Mustapic M Eitan E Werner JK Jr. Berkowitz ST Lazaropoulos MP Tran J. Plasma extracellular vesicles enriched for neuronal origin: a potential window into brain pathologic processes. Front Neurosci. (2017) 11:278. 10.3389/fnins.2017.00278 28588440 PMC5439289

[178] Karnati HK Garcia JH Tweedie D Becker RE Kapogiannis D Greig NH. Neuronal enriched extracellular vesicle proteins as biomarkers for traumatic brain injury. J Neurotrauma. (2019) 36:975–87. 10.1089/neu.2018.5898 30039737 PMC6444902

[179] Gruzdev SK Yakovlev AA Druzhkova TA Guekht AB Gulyaeva NV. The missing link: how exosomes and miRNAs can help in bridging psychiatry and molecular biology in the contextof depression, bipolar disorder and schizophrenia. Cell Mol Neurobiol. (2019) 39:729–50. 10.1007/s10571-019-00684-6 31089834 PMC11462851

[180] Rider P Carmi Y Cohen I. Biologics for targeting inflammatory cytokines, clinical uses, and limitations. Int J Cell Biol. (2016) 2016:9259646. 10.1155/2016/9259646 28083070 PMC5204077

[181] Blankenship K. The Top 20 Drugs by Global Sales in 2019 (2020). *Fierce Pharma, July 27, 2020*. Available online at: https://www.fiercepharma.com/special-report/top-20-drugs-by-global-sales-2019 (accessed January 11, 2021).

